# A comprehensive study of plasmonic mode hybridization in gold nanoparticle-over-mirror (NPoM) arrays

**DOI:** 10.1515/nanoph-2025-0437

**Published:** 2025-12-04

**Authors:** Raphael Gherman, Sacha Schwarz, Jean-François Bryche, Guillaume Beaudin, Alex Currie, Pierre Levesque, François Fillion-Gourdeau, Steve G. MacLean, Dominique Drouin, Serge Ecoffey, Paul G. Charette

**Affiliations:** 7321 Institut Interdisciplinaire d′Innovation Technologique (3IT), Université de Sherbrooke, 3000 Boulevard de l’université, Sherbrooke, J1K OA5 QC, Canada; Laboratoire Nanotechnologies Nanosystèmes (LN2)-IRL3463, CNRS, Université de Sherbrooke, INSA Lyon, École Centrale de Lyon, Université Grenoble Alpes, Sherbrooke, J1K 0A5 QC, Canada; Infinite Potential Laboratories, Waterloo, N2L 0A9, ON, Canada; INRS-Énergie, Matériaux et Télécommunications, Varennes, J3X 1S2, QC, Canada; Institute for Quantum Computing, University of Waterloo, Waterloo, N2L 3G1, ON, Canada

**Keywords:** plasmonic cavity, strong coupling, hybrid mode, nanoparticle-over-mirror, photoemission electron microscopy

## Abstract

Hybrid plasmonic systems that combine localized and propagative surface plasmons offer new opportunities for tunable light–matter interactions at the nanoscale. This paper provides the most comprehensive study to date of hybridization between gap localized surface plasmons (gap LSP) and diffraction-mediated propagative surface plasmon polaritons (SPP) in arrays of gold nanodisks over a mirror, part of the larger class of nanoparticle-over-mirror (NPoM) devices. By systematically mapping the hybrid mode dispersion as a function of array geometry over a large parameter space, we extract the coupling strength via a coupled oscillator model and reveal its dependence on key structural parameters, with gap thickness identified as the primary tuning factor. The resulting hybrid modes enhance the optical quality factor by nearly fivefold compared to classical LSP while maintaining strong near-field confinement, combining the advantages of their constituent modes. Dephasing times were measured with interferometric time-resolved photoemission electron microscopy (ITR-PEEM). Using a scalable lithography-compatible NPoM architecture that minimizes the optical index mismatch between the dielectric between the nanodisks and the gap material (Al_2_O_3_), we achieved the highest coupling strength (123 meV) and dephasing time range (23–50 fs) to date in NPoM arrays.

## 1 Introduction

Plasmonic cavities have the ability to confine and manipulate light at the nanoscale [1], enabling a broad range of applications in nanophotonics. These cavities exploit the collective oscillations of conduction electrons in metals to generate evanescent fields at metal–dielectric interfaces within extremely small mode volumes, typically 1–100 nm^3^ [1], [2]. Such confinement has been harnessed for biosensing [3], [4], perfect absorbers [5], [6], surface-enhanced Raman spectroscopy (SERS) [7], [8], emission enhancement [9], and strong coupling with quantum emitters [10], [11]. Localized surface plasmon (LSP) resonances in metallic nanoparticle-over-mirror (NPoM) structures play a central role in these effects [12]. In this architecture, Au or Ag nanoparticles (NPs) are deposited above a continuous metallic film separated by a nanoscale dielectric spacer. The near-field coupling between the NP and its mirror image produces strongly confined gap modes [13] due to opposite charge accumulation, analogous to dimer plasmons. Disk- or cube-shaped NPoMs can combine deep subwavelength confinement with enhanced radiative efficiency [14], making them promising systems for exploring the transition from weak to strong light–matter coupling, particularly with excitonic two-dimensional semiconductors [9], [15], [16], [17], [18], [19], [20], [21].

However, NPoM structures are usually fabricated via bottom-up methods such as random dispersion or self-assembly, which limits spatial control and introduces variability. Also, despite their high field confinement, the performance of individual plasmonic cavities is constrained by intrinsic losses [22]. Non-radiative damping from electron scattering limits quality factors in the visible range to Q ≈ 1–20 [2] and restricts resonance dephasing times to only a few femtoseconds [23]. To mitigate these losses, periodic arrays of NPs have been introduced, including NPoM arrays [24]. Periodicity enables long-range coupling and gives rise to collective resonances such as surface lattice resonances (SLRs) or propagating surface plasmon polaritons (SPPs) [25]. Fabrication methods for NPoM arrays are now controlled and reproducible, including top-down lithography [26], NP self-assembly into functionalized holes [27], and damascene chemical–mechanical polishing of gold developed by our group [28].

When the dielectric spacer thickness between the mirror and the nanostructures is <100 nm, incident light can couple to propagating SPPs at the mirror–dielectric interface through the in-plane momentum imparted by the array [25], [29], [30], [31], [32]. These SPP modes are often described as grating-coupled SPP-Bloch (or Bragg) modes, but for clarity, we refer to these modes simply as SPP in this article. Notably, when SPP and LSP gap modes spectrally overlap and spatially interact, they hybridize to form new modes [24], [25], [27], [33], [34], [35], [36], [37], [38]. The characteristic anti-crossing in the hybrid mode dispersion diagram is indicative of coherent energy exchange between the constituent modes, combining the strong confinement of LSPs with the extended coherence and narrow linewidths of SPPs. However, studies to date on such structures are mainly numerical and extensive studies based on fabricated structures of how geometry controls coupling strength, losses, and coherence are lacking.

In this work, we address this knowledge gap with a comprehensive study of hybrid plasmonic modes arising from the coupling between SPP and gap LSP in gold NPoM arrays. Using both numerical simulations and experimental measurements, we map hybrid mode dispersions across a wide parameter space of disk diameter, array pitch, and gap thickness. In addition, the proposed structure geometry improves on previous designs to achieve stronger coupling to incident light and mode hybridization. We interpret the hybridization mechanism in a bonding–antibonding framework, supported by charge and field distribution analyses, and independently demonstrate the contributions of the constituent modes with test structures that isolate individual contributions of SPP and gap LSP. Finally, we probe the temporal dynamics of hybrid and uncoupled modes at the femtosecond scale using interferometric time-resolved photoemission electron microscopy (ITR-PEEM), providing direct measurements of plasmon dephasing times over an unprecedentedly broad range. This comprehensive approach allows us to identify the relationships between the geometry and various performance metrics such as coupling strength, quality factor, and dephasing time, over a wide a range.

## 2 Results and discussion

**Figure 1:**
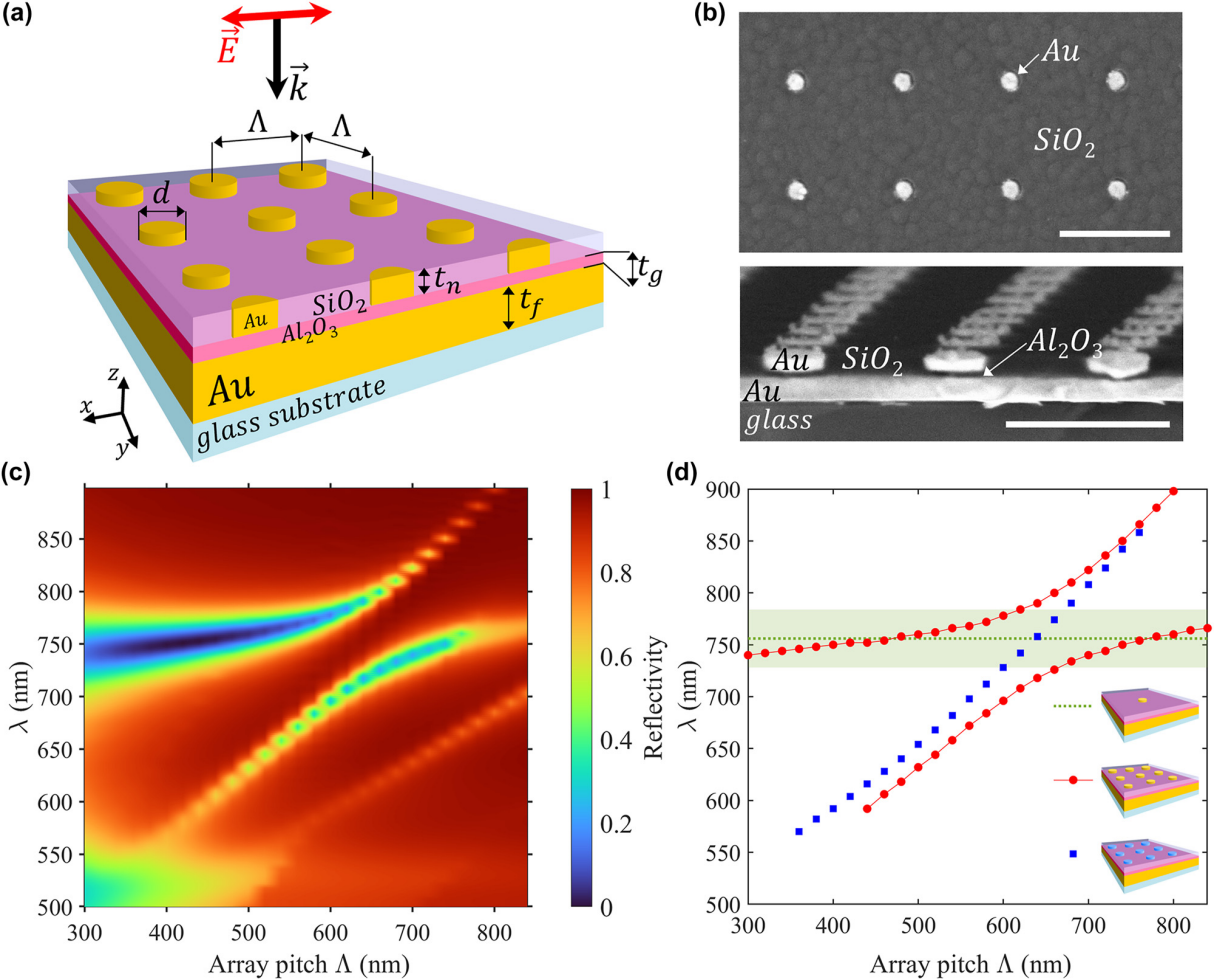
Hybridization between SPP and gap LSP modes in a gold NPoM array. (a) Schematic of the NPoM array structure, including illumination conditions: normally incident plane wave with linear polarization along the *x*-axis. (b) SEM images of a fabricated structure showing a surface view (top) and a cross-section view (bottom). Scale bars: 500 nm. Structural parameters: *t*_*g*_ = 18 nm, Λ = 500 nm, *d* = 83 nm (top image) and *d* = 180 nm (bottom image). (c) Simulated far-field reflection maps as a function of NPoM array pitch with gold nanodisk, at *t*_*g*_ = 18 nm and *d* = 90 nm. (d) Dispersion diagram of the hybrid modes. The green dotted line indicates the extinction cross-section maximum for a single NPoM with the green shaded region corresponding to the FWHM. The red and blue dotted lines represent the reflection minima for the gold NPoM and dielectric NPoM arrays, respectively. Corresponding reflectivity maps – both experimental and simulated – are provided in SI Figure A1, where *t*_*g*_ = 18 nm and *d* = 90 nm.

The NPoM arrays were both simulated and fabricated according to the geometry depicted in Figure 1a. The system consists of gold nanodisks embedded in silicon oxide (SiO_2_, thickness *t*_*n*_, refractive index 1.45) above a continuous gold film (mirror), separated by an aluminum oxide gap (Al_2_O_3_, thickness *t*_*g*_, refractive index 1.65). Fabrication was achieved via plasma etching followed by a lift-off process, as described in the *Methods and Fabrication* section. Excitation light is incident on the structure at normal incidence (except at 65° for comparison with ITR-PEEM near-field measurements, see below), with linear polarization aligned along the *x*-axis corresponding to one of the array’s principal periodic directions. Throughout this study, the gold film thickness (*t*_*f*_ = 100 nm) and SiO_2_ embedding layer thickness (*t*_*n*_ = 50 nm) are held constant across all simulations and fabricated devices. The effects of varying gap thickness (*t*_*g*_) and nanodisk diameter (*d*) are investigated below, particularly in relation to the mode behavior as a function of array pitch (Λ). Figure 1b presents scanning electron microscopy (SEM) images of a fabricated gold NPoM array. The nanodisks, surrounded uniformly by SiO_2_, exhibit high dimensional fidelity and homogeneity, which minimizes the parasitic broadening of the optical response related to material and geometry variability. Note that, compared to previous work by others [24], the geometry proposed here is advantageous because: (1) embedding the NPs in SiO_2_ (refractive index close to that of the alumina spacer) enhances the strength of the far-field resonance (see SI Figure A4); (2) the thick gold mirror (*t*_*f*_ > skin depth of gold in the visible range) ensures than only single-sided SPP at the mirror/spacer interface are supported which maximizes the overlap integral with the gap LSP modes.

The optical response of the structures is simulated in Figure 1c, which displays the reflectivity spectra of a gold NPoM arrays for different array pitches Λ (*t*_*g*_ = 18 nm, *d* = 90 nm). The resonance positions were determined by the minima in the simulated reflectivity spectra and plotted on Figure 1d, showing the mode dispersion as a function of array pitch Λ. Three configurations are compared: a single gold NPoM, a gold NPoM array (infinite periodic structure), and a dielectric NPoM array (non-metallic disks, infinite periodic structure), to distinguish how coupling manifests in the dispersion behavior of the hybrid modes. Reflectivity maps of the aforementioned structures are available in Supporting Information (SI) Figure A1. The faint horizontal resonance visible at shorter wavelengths (∼520 nm) and ∼300 nm pitch in Figure 1c corresponds to a higher-order localized plasmon resonance (quadrupolar gap mode, field maps not shown) which is not of direct interest in this study.

The green dotted line in Figure 1d corresponds to the resonance of a single gold NPoM identified by the extinction cross section maximum (see SI Figure A3). The material stack and geometry of the single NPoM are identical to the unit cells in the NPoM array, only the boundary conditions differ, as detailed in the FDTD section in *Methods and Fabrication*. This gap LSP resonance is characterized by a flat dispersion and a broad full width at half maximum (FWHM, green shaded area) due to losses (radiative and ohmic losses). For this mode, near-field coupling occurs between the nanodisk and its image in the mirror, resulting in a dimer-like behavior that we refer to as *gap LSP* throughout the paper. This resonance is significantly different from a nanodisk alone as the field is highly localized in the gap region between the disk and the mirror (see SI Figure A3).

The blue dotted line in Figure 1d indicates the simulated resonance position of a grating-coupled SPP, where the gold nanodisks in the array were replaced with dielectric nanodisks (refractive index 2.2) above the gold mirror. This configuration doesn’t support gap LSP because of the non-plasmonic nature of the nanodisks, thereby highlighting and isolating the SPP mode excited by the array. While the dielectric grating is less efficient for SPP excitation compared to its metallic counterpart, it provides a valuable reference for understanding pure SPP behavior.

The red dotted line in Figure 1d shows the dispersion of the hybrid modes in the gold NPoM array, with a distinct splitting centered at pitch of 640 nm at a wavelength of 756 nm. This anti-crossing behavior is a hallmark of strong coupling between the SPP excited by the grating and the gap LSP. This interaction results in the formation of two hybrid modes: a high-energy (short-wavelength) branch and a low-energy (long-wavelength) branch. Notably, the low-energy branch is consistent with the non-dispersive nature of the gap LSP for pitches below 550 nm, but becomes increasingly dispersive beyond 700 nm, gradually converging with the dielectric NPoM SPP dispersion. Conversely, the high-energy branch exhibits dispersive behavior at small pitches and flattens at larger pitches. This mode splitting within the hybridization region (540 nm < Λ < 740 nm) reflects the emergence of hybridized states resulting from coherent energy exchange between SPP and gap LSP modes. These hybrid modes were confirmed experimentally in reflection spectra (SI Figure A1). Furthermore, at larger pitches, a second anti-crossing is observed in simulations, attributed to the excitation of a higher-order SPP mode, which also interacts with the gap LSP (data not shown). The residual slope observed outside the anticrossing region arises from weak near-field coupling between adjacent NPoMs at small pitches for the low energy band, and from interaction with the higher-order SPP(1,1) mode at larger pitches for the high energy band.

To investigate the nature of the hybridization in gold NPoM arrays, we modeled by FDTD (details in *Methods and Fabrication*) the optical response of the structure at array pitches both inside and outside the hybridization region.

### 2.1 Modeled responses at array pitches outside the hybridization region

**Figure 2:**
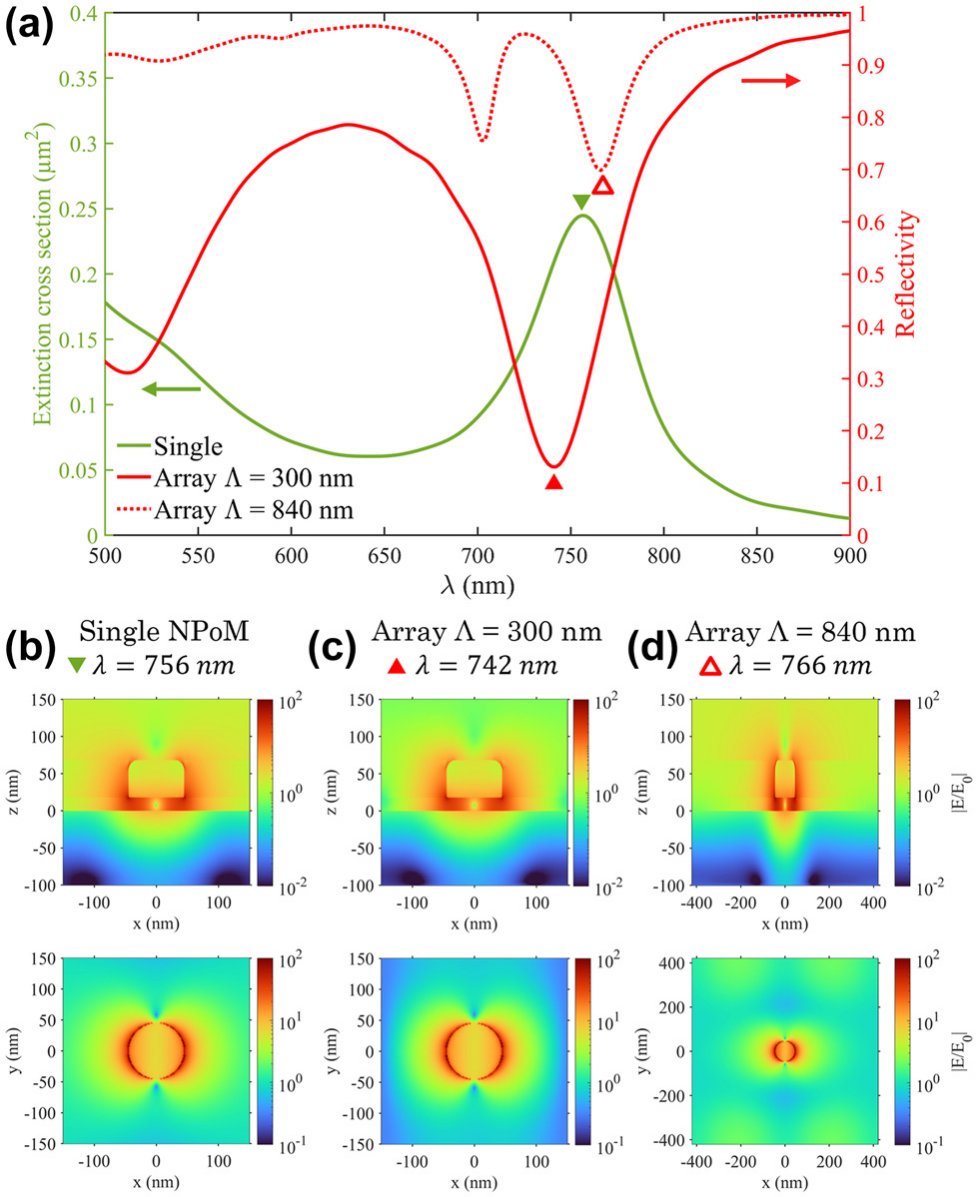
Modeled reflectivity from gold NPoM arrays at pitches outside the hybridization region, with *t*_*g*_ = 18 nm and d = 90 nm. (a) Extinction cross section spectrum for a single NPoM (green) and reflectivity spectra for gold NPoM arrays (red) for two different pitches (solid: Λ = 300 nm, dotted: 840 nm). (b–d) Corresponding electric field amplitude distributions at the resonant wavelengths shown in two planes: vertical cross-section through the center of the nanodisk (*y* = 0 nm, top panels) and horizontal plane located at the base of the nanostructures (*z* = *t*_*g*_, bottom panels).

**Figure 3:**
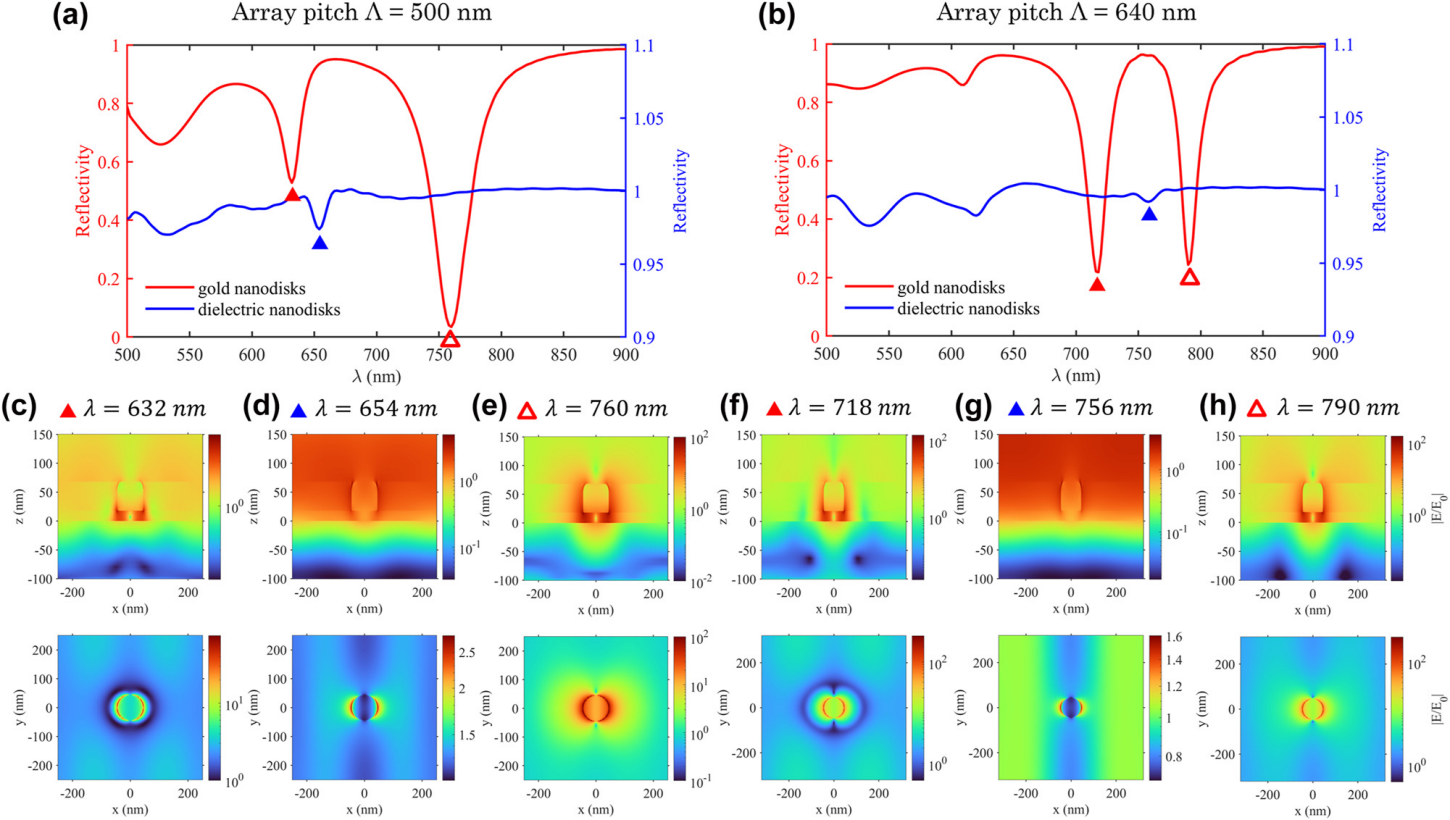
Modeled reflectivity from gold and dielectric NPoM arrays in the hybridization region. Data for array pitches at the edge (500 nm, detuned) and the center (640 nm, tuned) of the hybridization region, with *t*_*g*_ = 18 nm and *d* = 90 nm. (a–b) Reflection spectra of the NPoM array with gold nanodisks (red) and dielectric nanodisks (blue). (c–e,f–h) Corresponding electric field amplitude distributions at the resonance wavelength indicated in (a–b), shown in two planes: a vertical cross-section through the center of the nanodisk (*y* = 0 nm, top panels) and a horizontal plane located at the base of the nanostructures (*z* = *t*_*g*_, bottom panels).

Figure 2a shows the extinction spectra of a single NPoM structure, with a resonance at 756 nm corresponding to the gap LSP confined in the dielectric spacer between the nanodisk and the underlying gold film, as demonstrated earlier by the green dotted line in Figure 1d. This gap mode exhibits strong field confinement with an electric field amplitude enhancement of up to 10^2^. The corresponding electric field distribution is shown in Figure 2b (left column, top), clearly demonstrating localization within the gap. Additional analysis of the electric field components and charge distribution (SI, Figure B2) reveal a pronounced dipolar pattern in the E_z_ component, where charge accumulates predominantly at the base of the nanodisk, consistent with the near-field enhancement. While the fundamental gap plasmon (the l_1_ antenna mode) has a vertical dipole moment, the flat nanodisk over a mirror geometry supports transverse, waveguide-like cavity modes (s modes) within the metal-insulator-metal gap. At normal incidence, the in-plane polarized light efficiently excites the fundamental transverse mode (s_11_), which is characterized by charge accumulation at the nanodisk edges and a strong field in the gap. This mode, although transverse, is the dominant “gap mode” observed in the far-field for normal excitation. Numerical simulations show ≈95 % reflectivity dip at resonance (Figure 3a), and the charge-density maps (SI Figure B2) are consistent with the s_11_ transverse mode described in [39], [40], [41].

The reflectivity spectra for gold NPoM arrays at pitches below and above the hybridization region (Λ = 300 nm and Λ = 840 nm) are shown in Figure 2a, with resonance minima close to the single NPoM resonance wavelength. Note that the difference in resonance amplitude between the two cases is mainly due to a difference in fill factor. Despite the presence of the array, the near-field profiles in Figures 2c and d closely resemble those of the isolated NPoM structure, particularly in the immediate vicinity of the nanodisk. A difference of approximately −14 nm in the resonance position is observed between the single nanodisk and Λ = 300 nm case due to near-field interactions between adjacent nanodisks which slightly perturb the local optical environment. A difference of +10 nm is also observed between the single nanodisk and Λ = 840 nm due to a second resonance peak corresponding to the higher-order SPP mode (the field hot spots in the corners of the horizontal plane in Figure 2d/bottom are features of this resonance).

While these results suggest minimal influence from coupling with SPP in this regime, we further validated this hypothesis by examining the dependence of the resonance on the angle of the excitation light. Simulations at 10° angle of incidence (AOI) for both gold single NPoM and NPoM array with Λ = 300 nm (see SI Figure B1) showed negligible variations in the resonances, confirming that the mode is angle-independent – a hallmark of non-propagating, localized plasmons. In contrast, the presence of SPP modes would have introduced angular dispersion in the resonances [29]. This confirms that for pitches outside the hybridization region, the NPoM array mode resonance is decoupled from SPP modes and retains the characteristics of gap LSP.

### 2.2 Modeled responses at array pitches at the edge and the center of the hybridization region

Figure 3a shows the reflectivity spectra for the gold NPoM (red curve) and dielectric NPoM (blue curve) arrays for Λ = 500 nm, at the short wavelength end (or the small pitch end) of the hybridization region. Here SPP and gap LSP resonances are not yet fully tuned and there is a clear distinction between the high and low energy modes. The high energy mode (red curve, 632 nm) shows a resonance close to the SPP excited by the dielectric grating (blue curve, 654 nm), with a weaker amplitude (approximately 30 % in contrast difference) compared to the low energy mode (red curve, 760 nm).

In the lower panel of Figure 3c, corresponding to the high energy mode of the gold NPoM array at 632 nm, the field exhibits banded patterns between adjacent nanodisks which is characteristic of the standing wave resulting from the two counter-propagating propagating SPPs excited at normal incidence [25], [42]. A similar field distribution is observed in the dielectric NPoM array at 654 nm (Figure 3d, lower panel), reinforcing the identification of the high-energy mode as SPP-like. In contrast, the field map at 760 nm in Figure 3e (lower panel) displays strong confinement near the nanodisk that tapers off towards the edges of the simulation window, consistent with gap LSP-like behavior. These observations indicate that at Λ = 500 nm, the hybrid modes retain partial characteristics of their base components: the high-energy mode is primarily SPP-like, while the low-energy mode is predominantly gap LSP-like.

Note that the above analysis demonstrates that the mode hybridization is mediated by grating-coupled propagating SPPs rather than by SLRs [43], [44]. Though they also involve coupling offar-field illumination via a metal grating, SLRs result from the radiative coupling betweenlocalized plasmons of individual metal nanoparticles and are non-propagating in nature. Assuch, a purely dielectric grating cannot support SLRs, while it can excite propagating SPPs at anearby metal/dielectric interface, though inefficiently (blue trace in Figure 3a). Furthermore, thereflection minima in the dispersion diagram for dielectric grating-coupled SPPs correspondexactly to the bandgap in the hybrid mode dispersion diagram due to mode splitting (Figure 1d).Hence, though it is difficult to affirm without a doubt that SLRs play no role in this case, thearguments presented here make a compelling case for SPPs being the dominant modehybridization mechanism.

The reflectivity spectra in Figure 3b for Λ = 640 nm, at the center of the hybridization region, show two mode resonances with nearly equal amplitude, indicative of zero detuning and maximal hybridization. The SPP resonance of the dielectric NPoM array (blue curve) lies spectrally between the two hybrid modes of the gold NPoM array, confirming the anti-crossing behavior observed in Figure 1c and d. The field distributions in the lower panels of Figure 3f and h shows simultaneous features of both SPP and gap LSP modes: vertical field bands between nanodisks (SPP signature) strong field enhancement near the nanodisk edges (gap LSP signature). The evolution of hybrid mode behavior is further explored and discussed at larger array pitches in SI Figure C1 (Λ = 740 nm).

To gain deeper insight into the hybridization mechanism, we analyzed the charge distributions of different modes at the center of the hybridization region (Λ = 640 nm, see SI Figure C2) for the cases of the gold NPoM array (high and low energy hybrid modes at *λ* = 718 nm and 790 nm) and the dielectric NPoM array SPP mode (*λ* = 756 nm). In the dielectric NPoM array, the charges form alternating positive and negative bands in the *xy*-plane beneath the nanodisk, consistent with SPP field. For the metal NPoM arrays, the hybrid modes exhibit distinct bonding and antibonding charge configurations, analogous to molecular orbital interactions. In the high energy mode at 718 nm, charge accumulation patterns associated with the gap LSP (at the nanodisk edges) and the SPP (in vertical bands) are spatially out-of-phase. This results in a circular depletion ring approximately 100 nm from the disk center where the total electric charge is nil – resembling an antibonding orbital pattern. Conversely, in the low-energy mode at 790 nm, the charge distributions are aligned between SPP and gap LSP components, forming a merged pattern – analogous to a bonding orbital pattern. These bonding and antibonding configurations are schematically illustrated in SI Figure C2e. This analogy with molecular orbital theory provides a useful framework to interpret and identify plasmonic hybridization [45]. The final field redistributions result from the strong mode coupling in the NPoM array system.

### 2.3 Experimental study of the coupling

**Figure 4:**
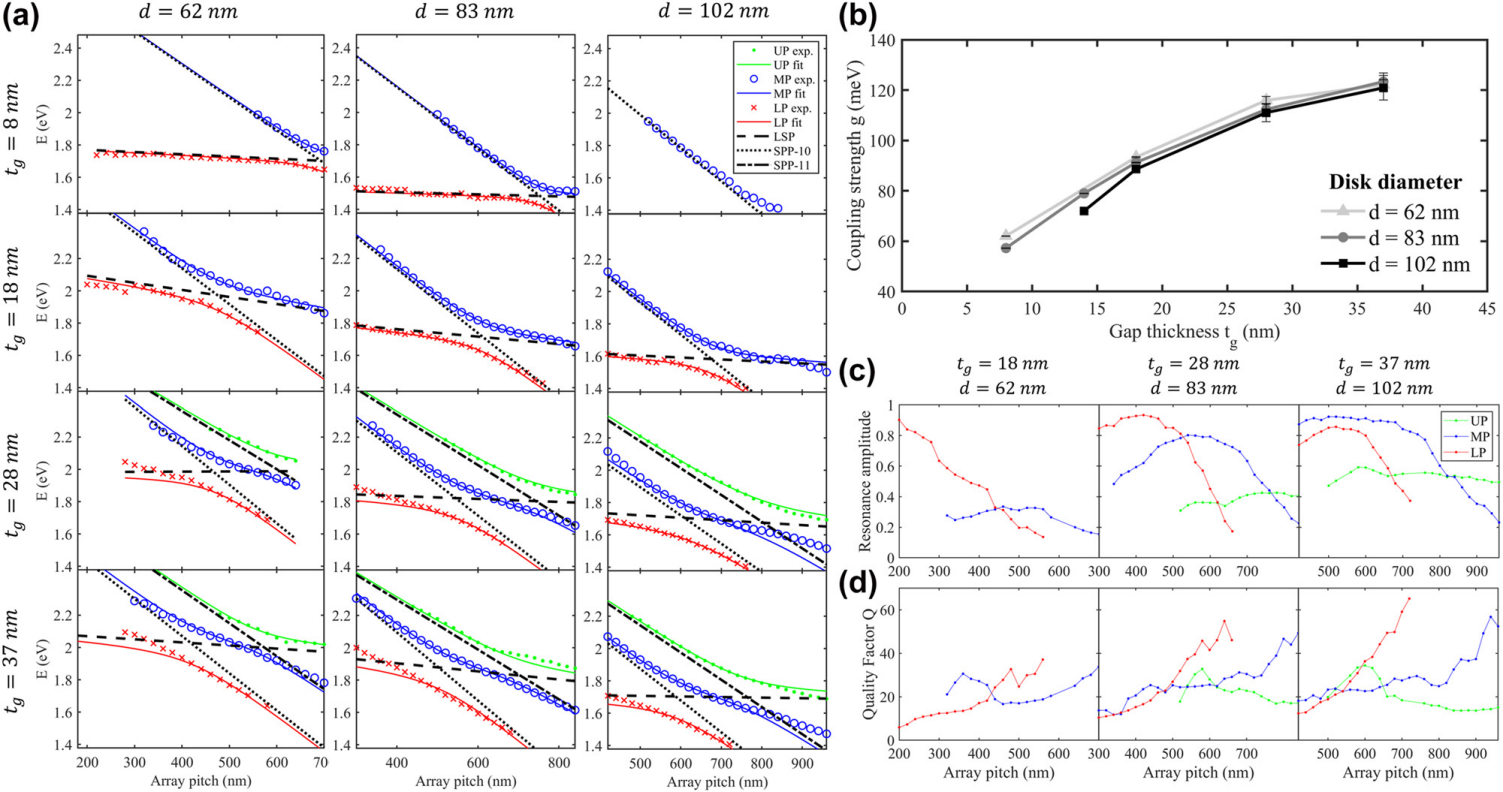
Coupling strength and hybrid mode dispersion in fabricated gold NPoM arrays. (a) Experimentally measured position of resonance peaks (markers) and fit with coupled oscillator models (lines). Blue circles, red crosses and green points are experimental measurements (UP, MP and LP modes) and continuous colored lines are corresponding dispersion curves predicted by the coupled oscillator models. The black continuous, dashed, and dotted lines trace the fitted dispersion of gap mode, lower-order SPP (SPP(1,0)) and higher-order SPP (SPP(1,1)) uncoupled modes, respectively. (b) Coupling strength between lower-order SPP (SPP(1,0)) and gap LSP as a function of gap thickness extracted from the fitting, for different nanodisk sizes. (c) Mode resonance strength (depth of the reflectance at resonance normalized by the nanodisk array density) and (d) optical quality factor Q of the hybridized modes as a function of array pitch. Experimental reflectivity maps for the extraction of the data are available in SI Figure D1.

To experimentally investigate mode hybridization and extract the relevant coupling parameters, we fabricated gold NPoM arrays across various combinations of array pitch, nanodisk diameter and gap thickness, and measured their reflectivity spectra as described in the *Methods and Fabrication* section. Resonance positions were extracted from the data (SI Figure D1) to build series of mode dispersion diagrams as a function of array pitch as shown in Figure 4a. Note that compared to the numerical results above calculated at fixed spacer thickness and disk diameter (*t*_*g*_ = 18 nm, *d* = 90 nm), the fabricated structures varied spacer thickness and disk diameter (8 nm ≤ *t*_*g*_ ≤ 37 nm, 62 nm ≤ *d* ≤ 102 nm). The experimental results confirmed the presence of the higher-order SPP mode (SPP(1,1)) mentioned above, in addition to the fundamental SPP mode (SPP(1,0)). The suffixes “10” and “11” refer here to the diffraction order used to excite the modes, either the first order along *x* only for “10” or the first order along both *x* and *y* for “11”. As shown below, three hybrid modes can be seen in the dispersion diagrams, classified by increasing energy: the “lower plasmon” (LP) and “middle plasmon” (MP) modes result from the hybridization between SPP(1,0) and the gap LSP, while the “upper plasmon” (UP) mode is due to coupling with SPP(1,1).

Coupled two- or three-oscillator models (SI Figure D2) were fitted to the data to quantify the coupling strength. These classical lossless models [46] accurately capture the observed trends as demonstrated by Figure 4a (wavelengths in nm were converted to energy *E* in eV to match the units of the coupled oscillator models in the context of light–matter coupling). The two-oscillator model accurately represents the LP and MP modes whereas the three-oscillator model is required by the influence of the SPP(1,1) mode for *t*_*g*_ larger than 20 nm to accurately model the UP mode. Indeed, the coupling between the SPP(1,1) and gap LSP modes significantly influences the interaction between the SPP(1,0) and gap LSP modes. At larger gap thicknesses (*t*_*g*_ > 20 nm), the SPP(1,0) and SPP(1,1) modes overlap with the red-shifted gap mode, resulting in a three-mode interaction mediated by the common near-field interaction. The dispersion was therefore fitted using a three-oscillator model (Figure 4a). Direct coupling between SPP(1,0) and SPP(1,1) remains negligible, consistent with previous studies [47], [43], and the coupling strength between SPP(1,0) and SPP(1,1) is set to zero in the hybridization model in SI Figure D2.

The dispersion curves and model fits in Figure 4a reveal several characteristics of the mode hybridization and its dependence on geometry:

– Gap LSP resonance redshifts with increasing nanodisk diameter and decreasing gap thickness, in agreement with prior studies [24], [25], [37].
– The SPP resonance redshifts with increasing array pitch and remains largely unaffected by changes in *d* or *t*_*g*_ *.*
– Consequently, the hybridization region shifts to higher pitch values for larger nanodisks (due to lower gap LSP energy) and to lower pitches for larger gap thicknesses (due to higher gap LSP energy).
– At larger gap thicknesses (*t*_*g*_ = 28 nm and 37 nm), the higher-order SPP mode (SPP(1,1)) becomes significant in the hybridization process. This mode couples with the gap LSP near the same pitch values where the SPP(1,0) + gap LSP hybridization occurs, thereby modifying the hybridization behavior.

The coupling strength *g* extracted from the fits to the coupled oscillator models is plotted in Figure 4b as a function of gap thickness for the different nanodisk diameters. The results indicate that mode coupling strength increases with gap thickness, with *g* reaching 123 meV at *t*_*g*_ = 37 nm, whereas nanodisk diameter has only a minor influence (the validity of data for *t*_*g*_ > 40 nm is discussed below). Note that coupling strength that *increases* with gap thickness would at first seem a surprising result (the trend is similar when normalizing by the mid-point energy at the zero-detuning point). Indeed, the electric field of the gap LSP mode is tightly confined beneath the nanodisks and thus the position of maximum field intensity shifts vertically as *t*_*g*_ increases [25]. In contrast, the SPP mode field amplitude decreases with distance from the mirror metal/dielectric interface. Consequently, the mode overlap between the two modes, and thus the coupling strength, would be expected to *decrease* as *t*_*g*_ increases. To understand this apparent discrepancy, we compute the overlap integral O between the two modes over the interaction volume *V*:

(1) $$\begin{array}{c} O=∭\varepsilon \left(\vec{r}\right)\left(\vec{E_{LSP}^{*}}\cdot \vec{E_{SPP}}\right)\left(\vec{E_{SPP}^{*}}\cdot \vec{E_{LSP}}\right)dV \end{array}$$

where $\vec{E_{LSP}}$ and $\vec{E_{SPP}}$ are the complex electric field distributions of SPP and gap LSP modes, respectively, and $\varepsilon \left(\vec{r}\right)$ is the material permittivity distribution. The overlap integral O does indeed decrease as *t*_*g*_ increases (see Figure D3b of the SI). However, when normalized by the total energies of the uncoupled modes, the *normalized* overlap integral $O_{\mathrm{norm}}$ given by:

(2) $$\begin{array}{c} O_{\mathrm{norm}}=\frac{O}{∭\varepsilon \left(\vec{r}\right)\left(\vec{E_{LSP}^{*}}\cdot \vec{E_{LSP}}\right)dV\times ∭\varepsilon \left(\vec{r}\right)\left(\vec{E_{SPP}^{*}}\cdot \vec{E_{SPP}}\right)dV} \end{array}$$

has the opposite behavior, as shown in SI Figure D3b, where $O_{\mathrm{norm}}$ increases with *t*_*g*_ in concordance with the experimental data. This would seem to indicate that though the coupling between the incident light and the constituent modes (SPP, gap LSP) is less efficient at increasing gap size (decrease in mode energy), the commensurate increase in mode hybridization results in a higher coupling strength overall. Detailed procedures for the mode overlap calculation and simulation parameters are given in SI Section D and Table D1. Note that for gap sizes *t*_*g*_ > ∼40 nm, the gap LSP gradually ceases to exist due to the finite near-field interaction range (∼30–40 nm) with the mirror and the mode transitions to a LSP mode around an isolated nanodisk, no longer hybridizing with the SPP (decreasing *g* in Figure 4b). At the larger gap sizes, the dispersion-less horizontal band associated with the gap LSP vanishes and the mode dispersion profile recovers its linear dependence with pitch, consistent with a pure SPP mode excited by the nanodisk array through diffractive coupling. This behavior is illustrated in SI Figure D4 showing reflectivity maps of NPoM arrays for various *t*_*g*_. These results suggest that there exists an optimal value for the gap thickness that maximizes coupling strength for a particular geometry.

The plots in Figure 4c show the mode “resonance strength”, quantified by the depth of the far-field reflectance at resonance normalized by the nanodisk array density, of the LP, MP, and UP modes, for different geometries. At the lower edge of the hybridization region (e.g.: Λ = 300 nm), the LP mode dominates. As Λ increases, the LP mode strength decreases while that of the MP increases. The decreasing LP mode strength reflects a transition from a pure gap LSP mode – characterized by strong field confinement and a large reflectivity dip – to a hybrid state incorporating SPP characteristics, which exhibit weaker field enhancement and lower reflectivity dip. Conversely, the MP mode evolves from a pure SPP mode with low strength, to a hybrid state with increasing gap LSP character and larger resonance strength. At ever increasing Λ, the MP mode strength eventually decreases, and the UP mode rises due to the appearance of the SPP(1,1) mode. Throughout this wide range in array pitch, the hybrid modes remain clearly distinguishable in the reflectivity measurements, with peak contrasts between 30 % and 95 %.

Figure 4d shows the reflectivity spectra optical quality factor *Q* = Δ*λ*/*λ* for the hybrid modes (Δ*λ* = FWHM) as a function of pitch for different geometries. Outside the hybridization region, the LP mode exhibits a relatively low Q-factor (∼10), primarily limited by intrinsic gap LSP losses. As hybridization with the SPP mode strengthens, the Q-factor of the LP mode increases to 20–30 in the hybridization region due the reduced non-radiative losses from the SPP component. Maximal values of Q reaching 50–60 are attainted beyond the hybridization region, for *d* = 102 nm and *t*_*g*_ = 37 nm. In contrast, the MP mode does not exhibit a decrease in Q-factor with increasing pitch. This behavior is possibly due to its hybridization with the SPP(1,1) mode, which becomes relevant at larger pitches and effectively compensates for the radiative and non-radiative losses associated with the gap LSP component. Hopfield-coefficient analysis (see Figure D5 in SI) for a representative geometry (*t*_*g*_ = 28 nm, *d* = 83 nm) confirms that the resonance amplitude decreases with SPP mode content of the hybrid mode, while the Q-factor increases with SPP mode content, consistent with the experimental trends.

The Q-factor of LSP modes is typically limited to 1–20 in the visible range [2]. Hybridization with SPP modes increases the Q-factor up to 60 in our case. However, this is still relatively low compared to SLR, which can reach Q-factors of several hundreds in the visible range [48], [49] and above 2000 in the near-infrared [50]. Nevertheless, the Q-factor alone does not fully capture a system’s potential for engineered light–matter interactions. The mode volume *V* must also be considered, as it plays a central role in determining the strength of these interactions [51]. For example, Purcell enhancement scales as *Q*/*V* and strong light–matter coupling efficiency as $Q/\sqrt{V}$ . Hybrid SPP – gap LSP modes in NPoM arrays exhibit very high field confinement in the nanometric gaps between the nanodisk and the mirror, a significant advantage over the comparatively weakly confined modes in SLR-based designs (see SI Figure A3b–c). Indeed, numerous studies have emphasized the significance of mode volume in light–matter interactions and its greater importance as a figure of merit relative to the Q-factor in certain applications [2], [51], [52].

### 2.4 Dephasing time measurements

To complement the far-field optical characterization, we employed ITR-PEEM to probe the near-field spectral and temporal dynamics of the hybrid plasmonic modes. PEEM is particularly well-suited for this purpose, as the photoemission (PE) signal arises from a nonlinear absorption process – typically involving the absorption of two or more photons with energy *ℏω* below the Fermi level to above the vacuum level – which provides direct access to the local electric field intensity [53]. We investigated the near-field properties of gold NPoM arrays by independently determining plasmon dephasing times in both time and frequency domains. Time-domain measurements were performed using ITR-PEEM [54], while the frequency-domain analysis is based on excitation-wavelength dependent PE (details in *Methods and Fabrication*).

**Figure 5:**
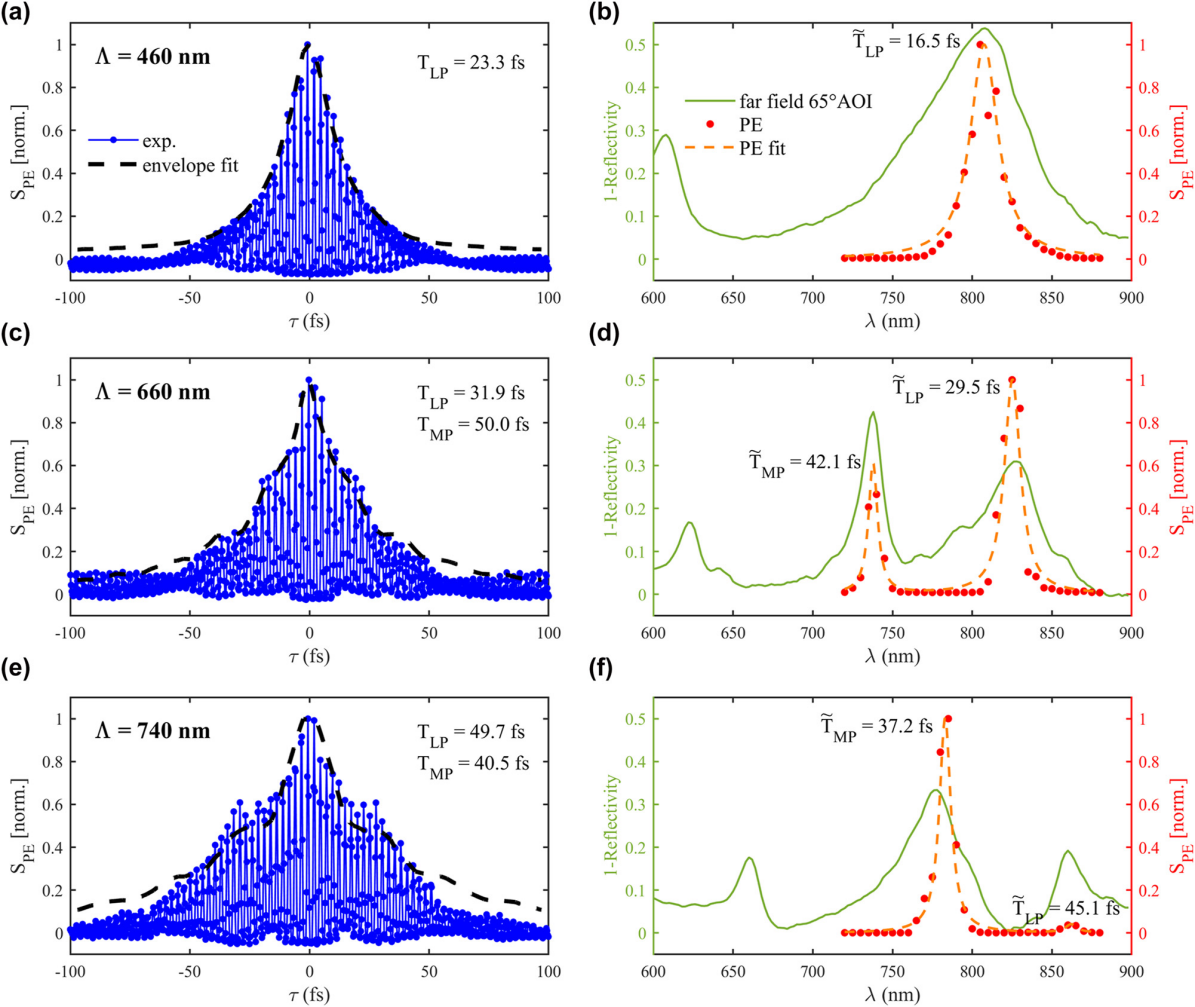
Near-field measurements in both time and frequency domain with *d* = 102 nm and *t*_*g*_ = 18 nm and various pitches Λ. (a,c,e) Spatially averaged PE yield (blue dotted line) for a given delay time *τ* from ITR-PEEM, and envelope fit calculated using ${\tilde{T}}_{LP}$ and ${\tilde{T}}_{MP}$ . By fitting (a) with a single and (b,c) with a coupled oscillator model (full data set available in SI Figure E4), the respective dephasing times *T*_LP_ and *T*_MP_ of the hybrid plasmonic modes are extracted. (b,d,f) Excitation-wavelength dependent PE yield (red dots), overlaid with the far-field optical measurement result at 65° AOI excitation in TE (solid green). By fitting the PE yield with a Lorentzian (dashed orange), the dephasing times ${\tilde{T}}_{LP}$ and ${\tilde{T}}_{MP}$ of the hybrid plasmonic modes are calculated using their PE spectral linewidth.

Figure 5 presents the near-field response in both time and frequency domain for a nanodisk size *d* = 102 nm and gap thickness *t*_*g*_ = 18 nm, corresponding to one of the diameter/gap configurations studied in Figure 4a. Figure 5a-c-e shows results from ITR-PEEM for pitches Λ = 460, 660 and 740 nm. Each data point represents a spatially averaged PE yield within a 11 µm field-of-view at given pulse delay *τ*, background-subtracted and flatfield corrected. Figure E2 in SI shows an example of the PEEM images used to extract the PE signal for different light excitations. The overall PE yield in the multiphoton regime is described by the expression:

(3) $$\begin{array}{c} S_{PE}\left(\tau \right)=\int_{-\infty }^{\infty }dt{\left|E_{loc}\left(t,\tau \right)\right|}^{2N} \end{array}$$

where we set the nonlinear order of the PE to *N* = 3, as discussed in [24]. The local field *E*_loc_(*t*, *τ*) is treated as a convolution of the incident field $E_{I}\left(t,\tau \right)$ with the temporal response function $R\left(t\right)$ of the NPoM array. Then, the overall PE yield $S_{PE}\left(\tau \right)$ is fitted by a model consisting of a single harmonic oscillator in the case of Λ = 460 and two coupled harmonic oscillators for Λ = 660 and 740 nm, respectively, and thus enables the extraction of dephasing times *T*_LP_ and *T*_MP_ [55], (see SI section E for a detailed derivation). Figure 5b-d-f presents the excitation-wavelength dependent PE yield result, which is proportional to the NPoM array absorption spectrum [56], by tuning the center wavelength of the incident field in the range *λ* = [720,880] nm in steps of 5 nm. The resulting distributions of spatially averaged PE yield at a given excitation wavelength are fitted by using a Lorentzian function with linewidth *γ*. Using the time-bandwidth product $\Delta \omega_{j}\cdot {\tilde{T}}_{j}=1$ where *j* ∈ {LP, MP} [57], the corresponding dephasing times ${\tilde{T}}_{\text{LP}}$ and ${\tilde{T}}_{\text{MP}}$ are extracted from the FWHM of the fitted Lorentzian Γ = 2*γ*.

In Figure 5b, 5d, and 5f, the electron emission peaks observed in PEEM align closely with the optical absorption resonances measured in reflectivity at 65° AOI. This agreement confirms that far-field absorption features correspond to near-field PE originating from regions of enhanced local field intensity. The strong local plasmonic fields in these regions increases the probability of electron emission via multiphoton absorption, enabling electrons to gain sufficient energy to overcome the work function and be detected in vacuum by PEEM. While the absolute amplitudes of the PEEM and reflectivity signals differ, a distinct trend is observed in their relative intensities through the hybridization region. A comparison of the FWHM reveals that the peaks from PEEM signal are narrower than their corresponding peak in far-field reflectivity spectra at 65°AOI. This broadening in reflectivity arises from ensemble averaging over a large number of nanostructures, which introduces inhomogeneities due to fabrication imperfections, grain boundaries, and variations in the local dielectric environment. Moreover, due to nonlinear scaling of multiphoton PE (∝ |E|^6^ for *N* = 3), the PEEM signal is highly biased toward nanostructures that generate the highest local fields. These high-field regions typically correspond to better-resonance, lower-damping geometries, leading to spectrally sharper features in PEEM compared to ensemble-averaged far-field reflectivity. Overall, these data suggests that near-field spectroscopy techniques are more effective for resolving peak splitting in the case of strong coupling between lossy plasmonic cavities and quantum emitters.

Dephasing times ${\tilde{T}}_{\text{LP}}$ and ${\tilde{T}}_{\text{MP}}$ extracted from Lorentzian fits of the PE spectra were used to calculate the envelopes (black dotted lines) plotted over of the ITR-PEEM signals (blue dotted line) in Figure 5a-c-e. Interestingly, these envelopes calculated from the PE spectral linewidth closely match the interferogram shape without any form of adjustment. These envelopes represent the maximum PE yield at each constructive interference delay, capturing the temporal decay of the modes. At Λ = 460 nm, the envelope closely follows a Lorentzian profile, while deviations from this profile emerge with increasing coupling, reflecting the complex dynamics of hybrid mode formation. In addition, the ITR-PEEM signal contains two key features: the slowly varying envelope related to mode dephasing (discussed above) and the fast oscillations due to the delayed pulses interferences. The analytical model described in SI Section E, based on either a single or coupled harmonic oscillator, successfully reproduces both features. Complete fits of the interferograms using the analytical model described in SI Section E are shown in SI Figure E4. The plasmonic dephasing times *T*_LP_ and *T*_MP_, displayed in Figure 5a-c-e, were extracted from this fitting procedure.

Analysis of the dephasing times across the hybridization region reveals the characteristic lifetimes of the constituent modes. The uncoupled gap LSP exhibits rapid decay, with dephasing times on the order of 15–20 fs, consistent with its known high loss due to ohmic and radiative damping, but already longer than classical LSP around single nanostructure [23]. Upon hybridization with the lower-order SPP, the LP dephasing time increases from 23.3 fs at Λ = 460 nm to 31.9 fs at Λ = 660 nm. Even beyond the hybridization region (Λ = 740 nm), the LP mode exhibits further temporal broadening, reaching 49.7 fs. This reflects the incorporation of low-loss SPP characteristics into the hybrid mode. Conversely, the MP mode, which initially exhibits a longer dephasing time of ∼50 fs at Λ = 660 nm, decreases to 40.5 fs at Λ = 740 nm, indicating a transition from SPP-like to gap LSP-like behavior, and a large modulation of plasmon dephasing time with the hybridization process. The ITR-PEEM results confirm both the dephasing time values and modulation behavior predicted by recent numerical studies [37].

## 3 Conclusions

Most studies to date on NPoM concern single isolated nanoparticles. An array configuration as studied here enables the hybridization of localized NPoM gap modes with propagating SPPs at the underlying metal/dielectric interface, a powerful tool to engineer optical hybrid modes properties. This work presented the most comprehensive study to date of strong coupling between diffraction-mediated SPP and gap LSP in NPoM arrays. Numerical modeling of mode dispersion as a function of geometric parameters, confirmed by experimental measurements, enabled the identification of spectral hybridization modalities between the constituent modes (SPP, gap LSP), while field distribution analyses clarified the spatial nature of the mode mixing. Results from single NPoM and dielectric NPoM test structures provided an in-depth understanding of the frequency-dependence of gap LSP and SPP contributions to the hybrid modes. ITR-PEEM enabled direct measurement of plasmon dephasing times with femtosecond resolution. We showed that LSP–SPP coupling can increase the quality factor of plasmonic modes by nearly fivefold, reducing dissipative losses while preserving strong near-field confinement. Gap thickness was found to be the dominant geometric parameter governing coupling strength, reaching an optimum value at larger gaps. The particular NPoM structure geometry proposed here, where the gold nanodisks are embedded in SiO_2_ to minimize the index mismatch with the gap material (Al_2_O_3_), combined with the extensive parameter space mapping in the numerical and experimental studies, enabled us to achieve the highest coupling strength and dephasing times to date with NPoM arrays, as demonstrated in Table 1.

**Table 1:** Comparison of coupling strength and dephasing time modulation with prior studies of SPP-gap LSP hybridization in gold NPoM arrays.

Reference	Study	Coupling strength (meV)	Dephasing time modulation (fs)
Yang et al. [24]	Experiment and simulation	78	6.5–13
Wang et al. [34]	Simulation only	93	Not given
Wang et al. [37]	Simulation only	88	23–166
This work	Experiment and simulation	123	23–50

Finally, the proposed NPoM array design is high scalable with a finely tunable lithography-compatible architecture. As such, gold NPoM arrays are highly promising for cavity quantum electrodynamics, ultrafast plasmonics, nonlinear optics, and nanoscale sensing. More specifically, this architecture is a natural pathway toward room-temperature integration with quantum emitters (2D semiconductors, quantum dots, or molecules) that would enable for both absorption and emission enhancement through near-field coupling, at the same time, and in significantly different spectral ranges. Hybridization between localized and propagating plasmons offers a direct route toward strong light–matter coupling, where delocalized optical fields coherently interact with localized emitters [58], [59], [60].

## 4 Methods and fabrication

### 4.1 Gold NPoM array fabrication

The gold NPoM arrays were fabricated by a lift-off method previously published by our group [28]. Briefly, using an Intelvac e-beam evaporation system, 100 nm thick Au layers with 2 nm Ti adhesion layers were deposited on glass BK7 substrates. A thin gap layer of Al_2_O_3_ was then deposited using a Plasmionique SPT320 magnetron sputtering system, followed by the SiO_2_ PECVD film at 250 °C with a Benchmark 800-II plasma processing tool. E-beam lithography was performed using a 200 nm thick ARP-6200 CSAR positive resist with Raith EBPG-5200 tool at 100 keV. The exposed resist was developed in MIBK for 1 min at room temperature. Etching of SiO_2_ was then achieved using CF4 based chemistry with an inductively coupled plasma process [61] in an STS Advanced Oxide Etching DRIE system. Because of the high selectivity of the plasma process (1/6), the alumina serves as an etch stop layer to control the gap thickness. The same resist was used to deposit 50 nm Au in the SiO_2_ wells with the same e-beam evaporation tool, without a Ti adhesion layer. The samples were then immersed for lift-off in acetone for 2 h and sonicated for 1 min, followed by cleaning with isopropanol and water. Areas of 500 × 500 µm^2^ gold NPoM arrays were fabricated with different nanodisk size and array pitch across a 6 × 6 mm^2^ area on the substrate.

### 4.2 Far-field reflectivity measurements

Far-field optical measurement of the array response was performed with a reflection setup in the visible range. Light emitted by a halogen lamp passed through a Horiba IHR 320 monochromator and a linear polarizer (polarization parallel to the substrate plane) incident onto the sample at normal incidence. The reflected light was routed by a nonpolarizing beam splitter and collected by a CMOS camera at each wavelength selected by the monochromator. Spectra were extracted by averaging the signal level of the image over the structured areas (500 × 500 µm^2^ arrays of nanodisks) and normalizing by the average signal level in the unstructured areas, at each excitation wavelength.

### 4.3 Near-field photoemission measurements

The plasmonic response of the arrays was probed in the near-field using multiphoton photoemission microscopy (PEEM; NanoESCA from Focus/Scienta Omicron) by means of two independent fs-laser systems. The excitation-wavelength-dependent PE was measured with fs-pulses width of less than 100 fs from a Spectra-Physics Ti:Sa oscillator Tsunami, while ITR-PEEM was realized with a Novanta Ti:Sa oscillator Venteon Ultra with dispersion compensated fs-pulses width of 6 fs. In both cases, the incident light interacted with the array at a grazing angle of 65° to the sample normal in TE configuration (zero electric field component out of the plane of the nanodisks array), forming a spot size of 120 µm diameter on average. For ITR-PEEM, the pulses were directed through a balanced Mach–Zehnder interferometer (MZI), generating interfering TE-polarized few-cycle pulses that illuminate the NPoM array. Group delay dispersion (GDD) within the system is managed using pairs of chirped mirrors and fine adjustments from ultrathin glass wedges. This dispersion compensation is calibrated via a frequency-resolved optical gating (FROG) setup at the second MZI output (see SI Figure E1). For both spectral and temporal measurement, the resulting PE at fixed kinetic energy selection was spatially averaged over a field-of-view of about 11 µm with a dwell time of 10 s and 400 ms per image, respectively, background-subtracted and flatfield corrected.

### 4.4 FDTD simulations

Electromagnetic simulations of the nanostructured plasmonic arrays were performed with the Ansys Lumerical FDTD software. For the NPoM arrays, periodic boundary conditions were applied in the *x* and *y* directions in the film plane at the edges of the simulation window. For single NPoM structures, perfectly matched layers (PML) were applied in the *x* and *y* directions at the edge of the simulation window and a total-field scattered-field approach was used with a plane-wave source. PML boundary conditions were applied in the *z* direction (perpendicular to film plane) at the edges of the simulation window for both models. The optical index of gold was based on experimental data acquired from a range of Au films deposited in our facilities. Modeled reflection values were normalized by results from the same model without nanostructures for comparison with experimental results. Replication data contains the models for every simulation result in this article.

## Supplementary Material

Supplementary Material Details
