# Supplementary material for: A comprehensive study of plasmonic mode hybridization in gold nanoparticle-over-mirror (NPoM) arrays
Source: Nanophotonics. 2025 Dec 4;14(27):5347–61. doi: 10.1515/nanoph-2025-0437 (PMC12717898; doi:10.1515/nanoph-2025-0437)
Supplement: Supplementary file 1 — Supplementary Material Details [file j_nanoph-2025-0437_suppl_001.docx]

Supporting Information (SI)

A Comprehensive Study of Plasmonic Mode Hybridization in Gold Nanoparticle-over-Mirror Arrays: Manipulation of Coupling and Dephasing Time

Raphael Gherman^1,2^, Sacha Schwarz ^3^, Jean-François Bryche^1,2^, Guillaume Beaudin^1,2^, Alex Currie^3,4^, Pierre Levesque^3^, François Fillion-Gourdeau^3,4^, Steve G. MacLean^3,4,5^, Dominique Drouin^1,2^, Serge Ecoffey^1,2^, Paul G. Charette^1,2^

^1^Institut Interdisciplinaire d′Innovation Technologique (3IT), Université de Sherbrooke, 3000 Boulevard de l'université, Sherbrooke, J1K OA5 Québec, Canada

^2^Laboratoire Nanotechnologies Nanosystèmes (LN2)-IRL3463, CNRS, Université de Sherbrooke, INSA Lyon, École Centrale de Lyon, Université Grenoble Alpes, Sherbrooke, J1K 0A5 Québec, Canada

^3^Infinite Potential Laboratories, Waterloo, Ontario, Canada, N2L 0A9

^4^INRS-Énergie, Matériaux et Télécommunications, Varennes, Québec, Canada J3X 1S2

^5^Institute for Quantum Computing, University of Waterloo, Waterloo, Ontario, Canada, N2L 3G1

# SPP – gap LSP hybridization


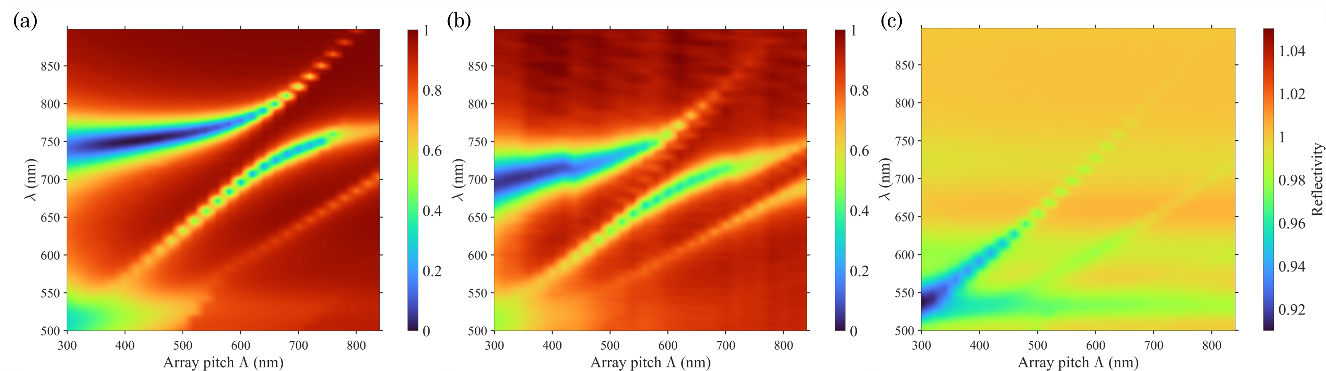


Figure A1: Simulated and experimentally measured far-field reflection maps as a function of NPoM array pitch. (a,c) Simulated map of array (a) with gold nanodisk and (c) with dielectric nanodisks, with t_g_ = 18 nm and d = 90 nm. (b) Experimental reflection map measurement for fabricated gold NPoM array, with t_g_ = 18 nm and d = 83 nm.

In the experimentally measured reflection map shown in Figure A1b, two types of spectral discontinuities are observed as the array pitch varies. The first type of discontinuity appears consistently at each pitch transition along the x-axis and is the result of a combined effect: a coarse sweep of pitch values and a high level of discretization in the colormap. This type is most noticeable when the resonance is sharp, and the position of the mode varies strongly; it also appears in the simulated reflection maps. The second type of discontinuity, seen only at pitch transitions of Λ = 420-440 nm and Λ = 700-720 nm in Figure A1b, is absent in the simulated map. This effect can be explained by the reflectivity measurements taken from different 500 × 500 µm² nanostructured areas spatially separated on the substrate. The large spatial separation across the substrate between areas with close pitch values (as in the cases of Λ = 420–440 nm and Λ = 700–720 nm) results in this discontinuity, which is likely due to slight non-uniformities in gap thickness across the substrate. This non-uniformity shifts the position of the gap LSP resonance without significantly affecting the SPP mode. Figure A2 quantifies this wavelength shift as the gap thickness varies across a narrow range of ±2 nm. If we assume that the fabrication process, which involves Al_2_O_3_ deposition and etching (for the gap definition), has a non-uniformity of 10% across the substrate, we expect a gap uncertainty around ±4 nm. This uncertainty would induce spectral shifts larger than the simulated one in Figure A2. This effect is also observed in the reflectivity maps in Figures D1 and D4.


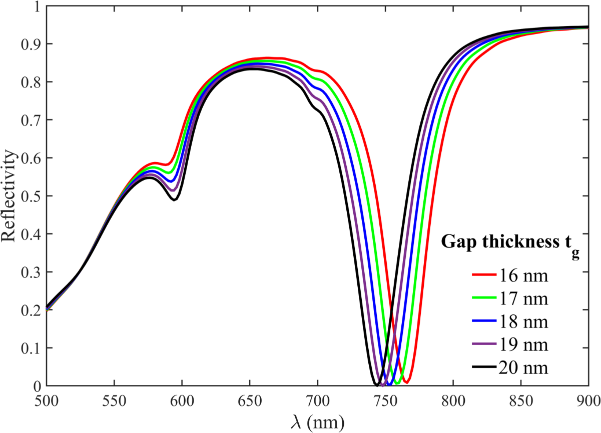


Figure A2: Simulated reflection spectra for different gap thickness t_g_ of NPoM array, with Λ = 440 nm and d = 90 nm, to show the high sensitivity of the gap LSP resonance position with t_g_, justifying the band discontinuities on the experimental map in Figure A1b due to non-homogeneity at the substrate scale of Al_2_O_3_ final thickness.


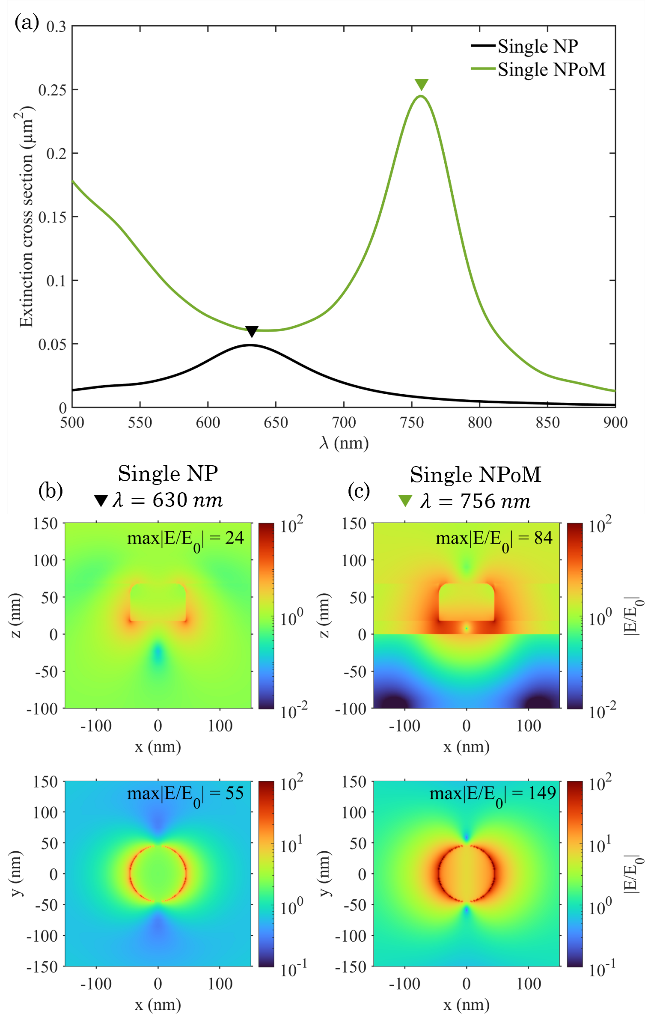


Figure A3: (a) Simulated extinction cross section spectrum for a gold single NP (black) and single NPoM (green), with t_g_= 18 nm and d = 90 nm. (b-d) Corresponding electric field amplitude distributions shown in two planes: vertical cross-section through the center of the nanodisk (y = 0 nm, top panels) and horizontal plane located at the base of the nanostructures (z = t_g_, bottom panels).


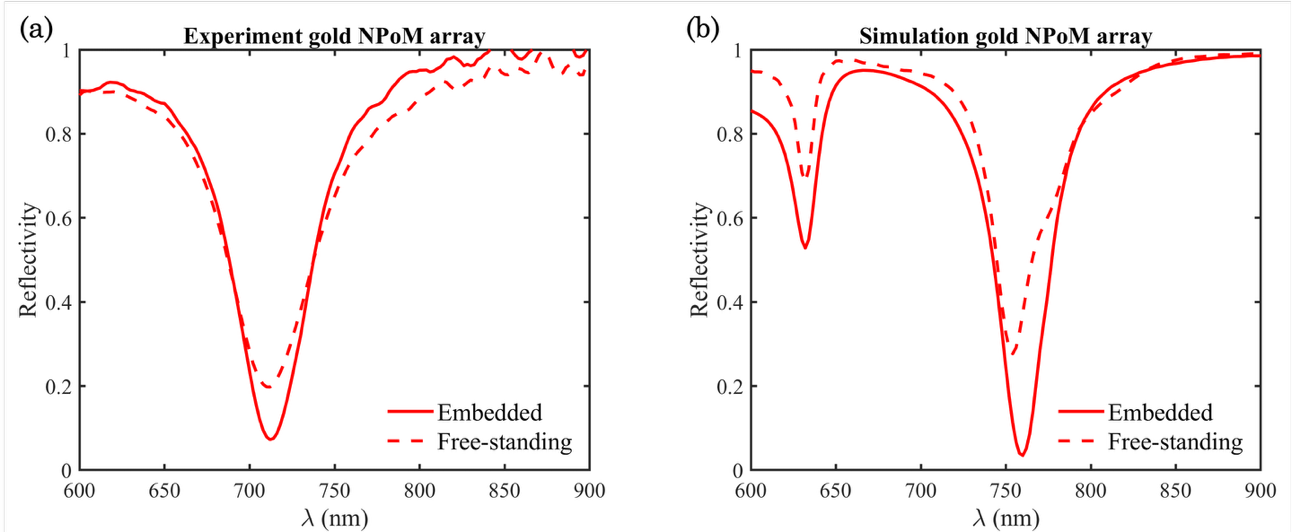


Figure A4: Effect of the embedding layer on plasmonic resonance. (a) Experimental and (b) simulated reflection spectrum of gold NPoM array, for embedded (solid lines) and free-standing (dotted lines) nanodisks. Here tg = 20 nm, and nanodisk diameter and array pitch are chosen to have spectrally close resonances for the embedded and the free-standing case. Geometries for experimental and numerical case are different, thus spectra are not comparable.

In Figure A5, we show the output of a reflectivity measurement at 650 nm wavelength for one sample containing two nanodisk size and series of array pitch to plot the full dispersion of the hybrid mode. Each square is 500 × 500 µm² array of NPoM that darkens when the resonance energy is matched. Spectra extracted from similar geometry but different locations on the 500 × 500 µm² array demonstrate high level of structure homogeneity, through the optical properties.


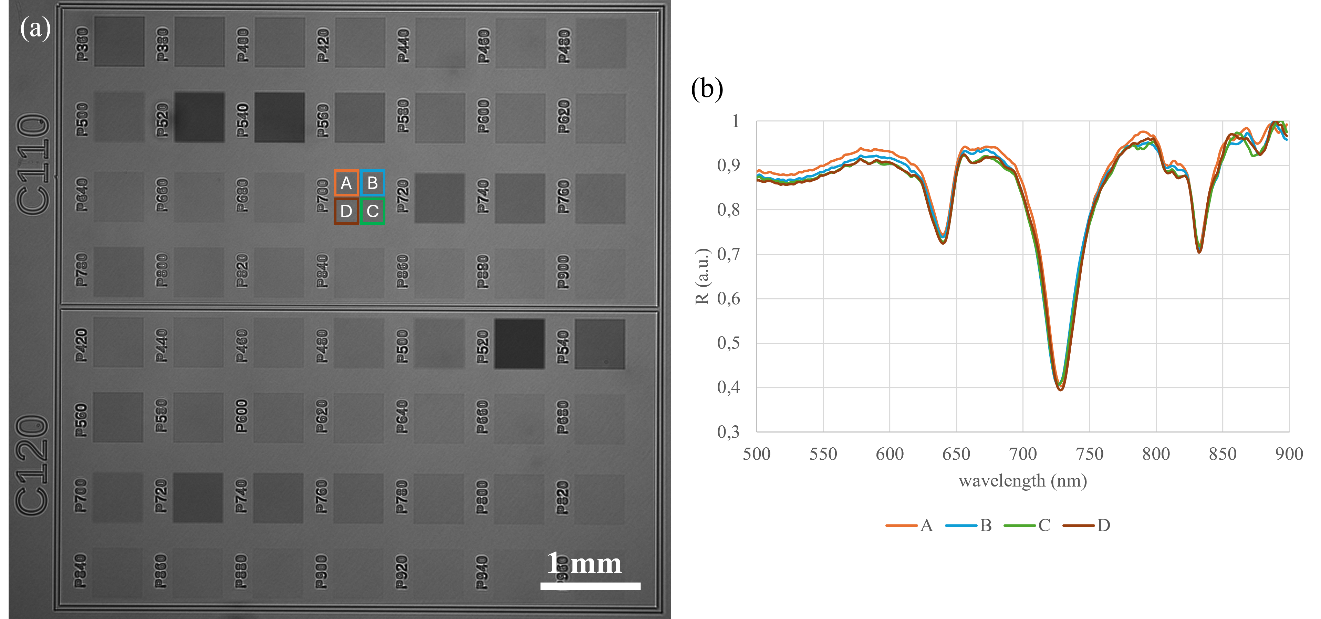


Figure A5: Reflectivity measurement (a) image at 650 nm excitation wavelength, showing multiple 500 × 500 µm² arrays of NPoM for various array pitches (P = pitch) and for two nanodisk sizes (b) Superposition of four spectra extracted from areas with the same geometry A, B, C, D.

# Modeled responses outside the hybridization region


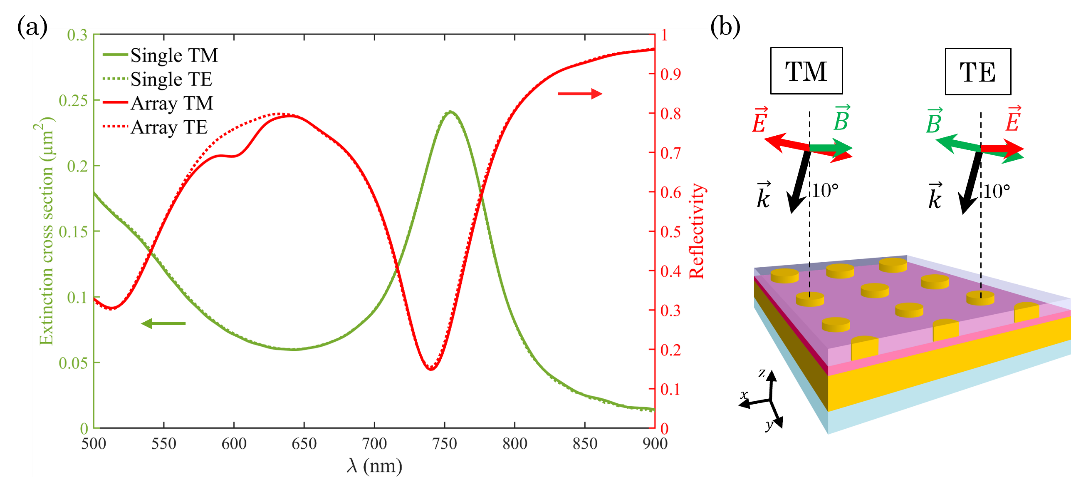


Figure B1: Simulated extinction cross section spectra of NPoM alone (green) and reflection spectra of gold NPoM array (red), with Λ = 300 nm, t_g_ = 18 nm and d = 90 nm. Light excitation at 10° angle of incidence in TE (red solid) and TM (red dotted) configurations, as depicted in (b).


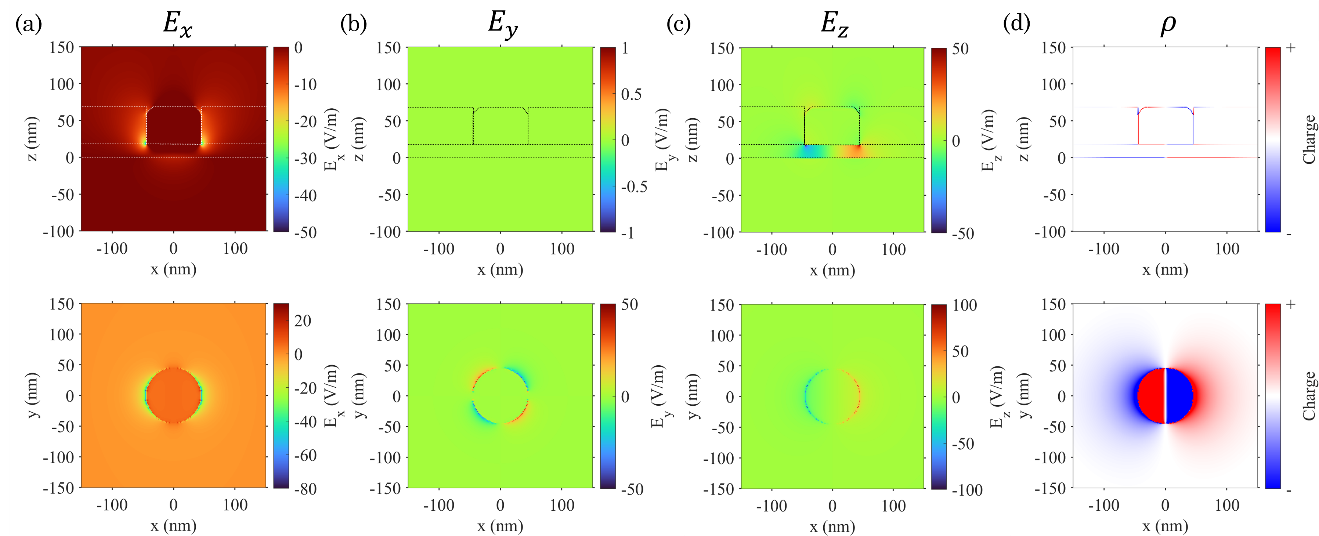


Figure B2: Simulated (a-c) electric field components and (d) charge distribution shown in two planes: vertical cross-section through the center of the nanodisk (y = 0 nm, top panels) and horizontal plane located at the base of the nanostructures (z = t_g_, bottom panels), at resonance of gap LSP in the single NPoM configuration (λ = 756 nm, t_g_ = 18 nm; d = 90 nm).

# Modeled responses in the hybridization region

At Λ = 740 nm, at the long wavelength end of the hybridization region, the spectral alignment between the low energy hybrid mode and the SPP mode of the dielectric array becomes apparent. Notably, the mode characteristics invert compared to Λ = 500 nm (see Figure 3a in main manuscript): the high energy hybrid mode now shows stronger gap LSP-like behavior, while the low energy mode becomes more SPP-like, both in spectral position and field distribution.


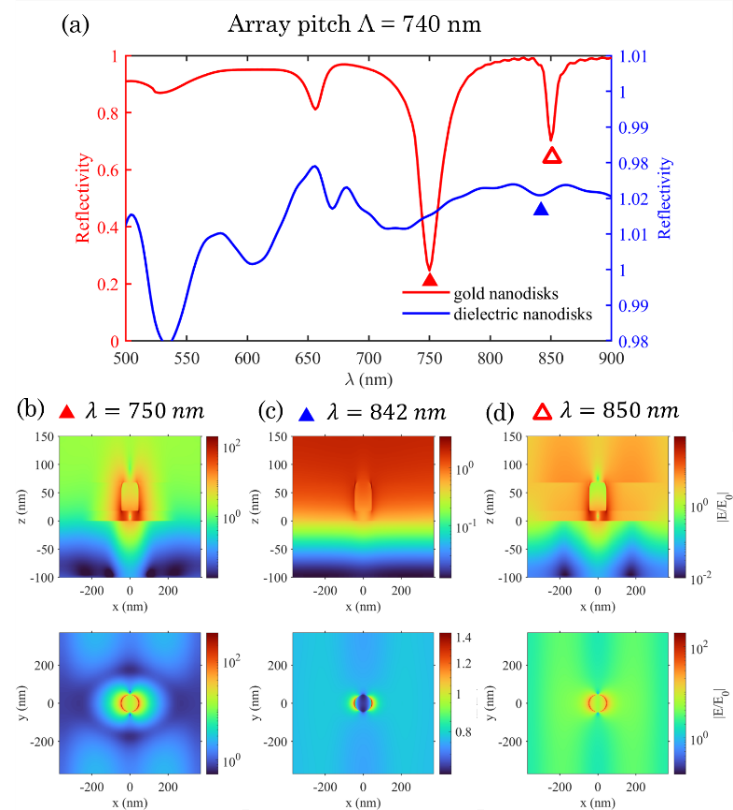


Figure C1: (a) Simulated reflection spectra of the NPoM array with gold nanodisks (red) and dielectric nanodisks (blue), with t_g_ = 18 nm and d = 90 nm. (b-d) Corresponding electric field amplitude distributions at the resonance wavelength indicated in (a), shown in two planes: a vertical cross-section through the center of the nanodisk (y = 0 nm, top panels) and a horizontal plane located at the base of the nanostructures (z = t_g_, bottom panels).


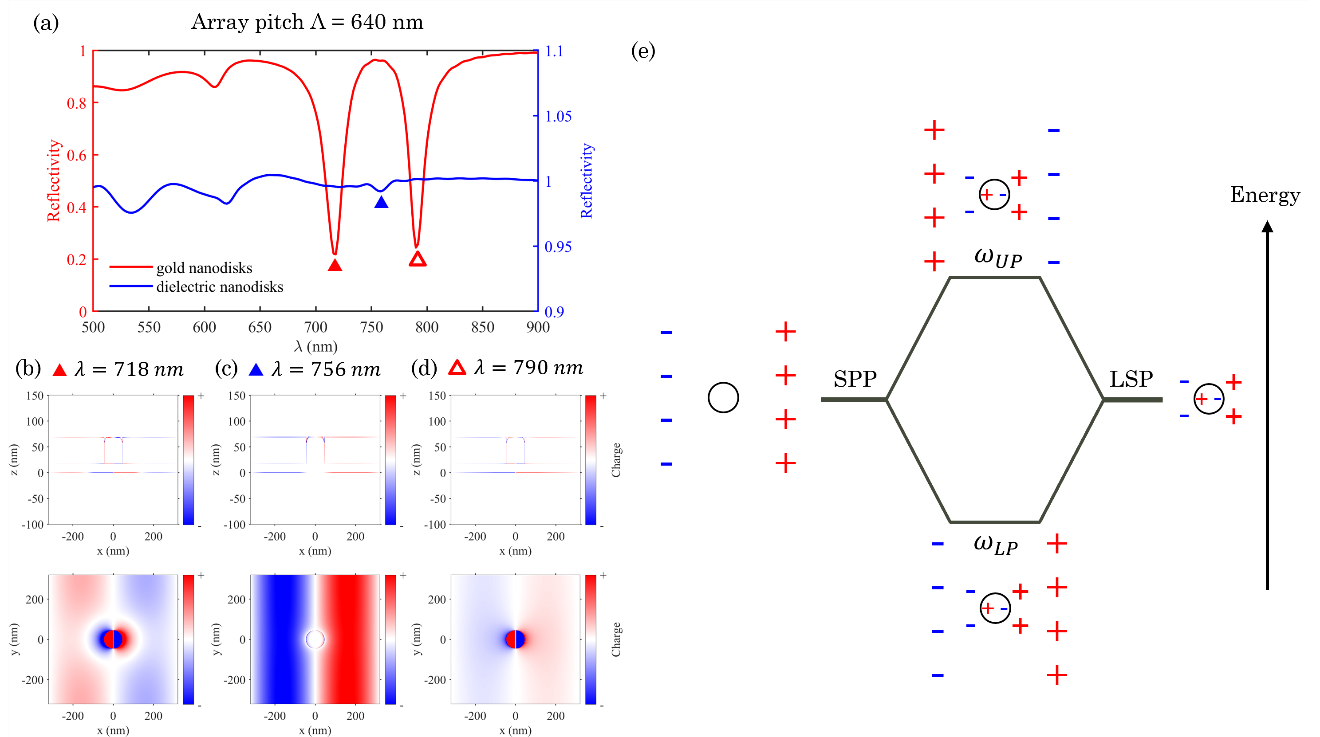


Figure C2: (a) Simulated reflection spectra of the NPoM array with gold nanodisks (red) and dielectric nanodisks (blue), with t_g_ = 18 nm and d = 90 nm. (b-d) Corresponding electric charge distribution at the resonance wavelength indicated in (a), shown in two planes: a vertical cross-section through the center of the nanodisk (y = 0 nm, top panels) and a horizontal plane located at the base of the nanostructures (z = t_g_, bottom panels). (e) Diagram of bonding (low energy, LP) and anti-bonding (high energy, UP) modes to explain charge distributions for the two hybridized mode gap LSP + SPP.

# Experimental study of the coupling


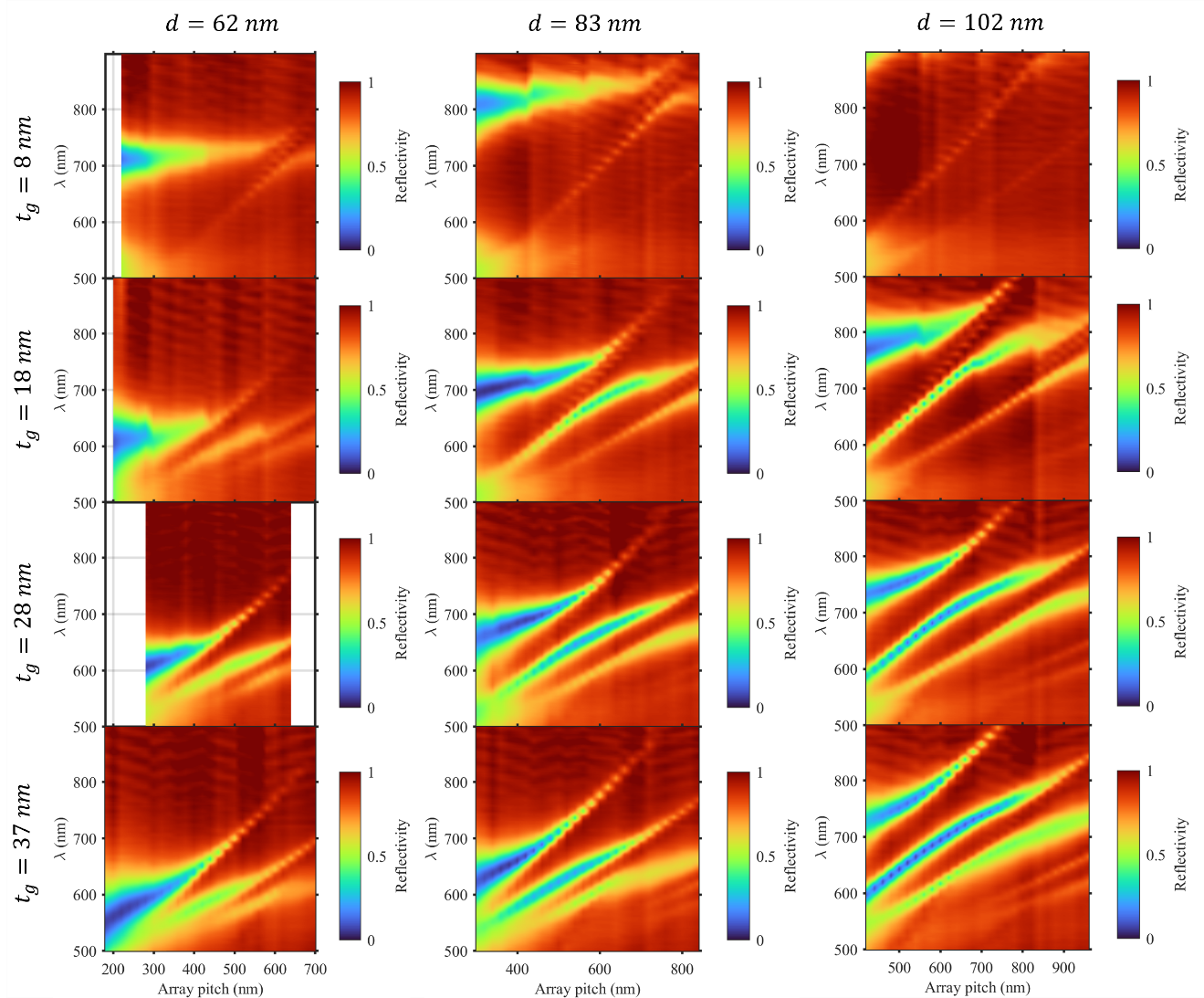


Figure D1: (a) Experimental measurements of reflection spectra as a function of array pitch used for the fit with coupled oscillator models in figure 4a, for different gap thicknesses and nanodisk diameters.


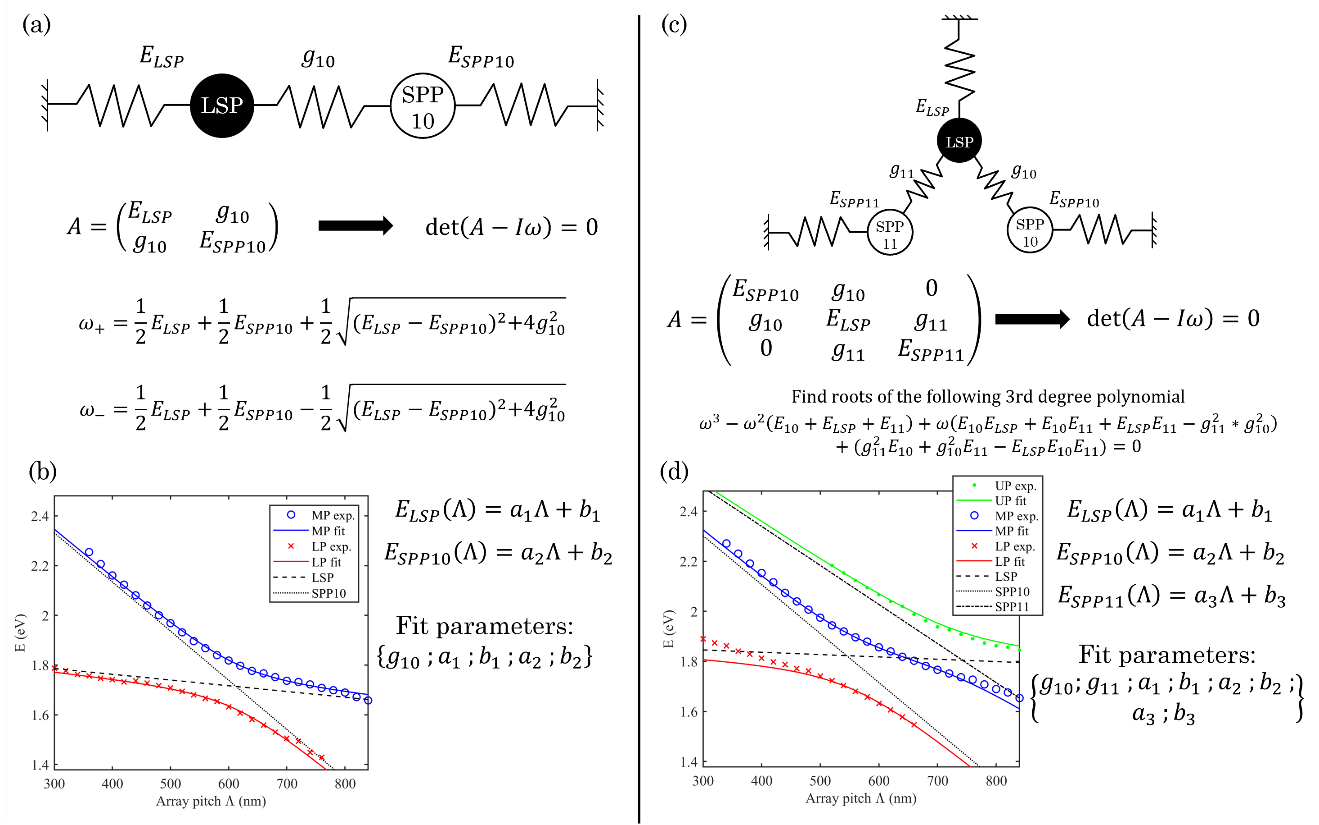


Figure D2: Classical (a-b) two and (c-d) three oscillator coupled oscillator models. (a,c) Theoretical calculation of the eigenfrequency for the hybrid modes and (b,d) fitting procedure to the experimental data with linear approximation of the non-coupled mode dispersion. (b) and (d) are extracted from Figure 4 with (d = 83 nm; t_g_ = 18 nm) and (d = 83 nm; t_g_ = 28 nm), respectively.


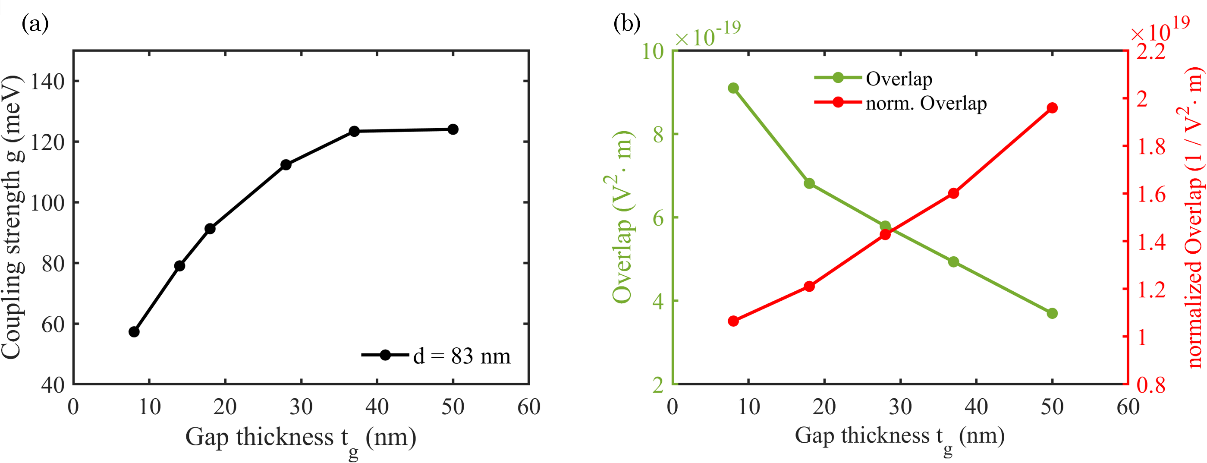


Figure D3: Mode overlap integral calculation. (a) Experimental coupling strength between gap LSP and SPP (low-order SPP) modes as a function of gap thickness, where d = 83 nm. (b) Calculated mode overlap and normalized mode overlap integrals based on field distributions extracted from FDTD simulation (model parameters detailed in Table D1).

We briefly outline the methodology used to calculate the mode overlap presented in Figure D3. The calculation is based on equations (1) and (2) from the manuscript using the electric field distributions obtained from FDTD simulations. To model the gap LSP mode, we simulated a single gold NPoM structure. The gap LSP resonance was identified from the reflectivity spectrum, and the corresponding three-dimensional electric field distribution was extracted within a simulation volume matching the array pitch used in the SPP model. This volume extends vertically from the base of the gold mirror to approximately 100 nm above the top of the nanodisk (total height: 300 nm). A similar procedure was applied to extract the SPP mode field using a dielectric NPoM array model. The resonance wavelength of the SPP mode was identified from its reflectivity spectrum, and the corresponding field distribution was obtained from the same spatial region as used for the gap LSP. The nanodisk diameter was set to 74 nm to match the position of the gap LSP out of the hybridization region. The array pitch in the dielectric NPoM array model was selected based on the center of the hybridization region, as determined from the fit of the hybrid mode dispersion using the coupled oscillator model shown in Figure 4 of the main text. A summary of the simulation parameters is provided in Table D1.

| **t_g_ (nm)** | **Gap LSP single gold NPoM simulation** | **Nanodisk diameter (nm)** | **Gap LSP resonance position (nm)** | **SPP dielectric NPoM array simulation** | **Array pitch (nm)** | **SPP resonance position (nm)** |
| --- | --- | --- | --- | --- | --- | --- |
| 8 |  | 74 | 832 |  | 750 | 814 |
| 18 |  | 74 | 721 |  | 610 | 734 |
| 28 |  | 74 | 681 |  | 540 | 702 |
| 37 |  | 74 | 660 |  | 500 | 692 |
| 50 |  | 74 | 684 |  | 460 | 678 |

Table D1: Simulation parameters of the FDTD models, used for the mode overlap calculation in Figure D3.


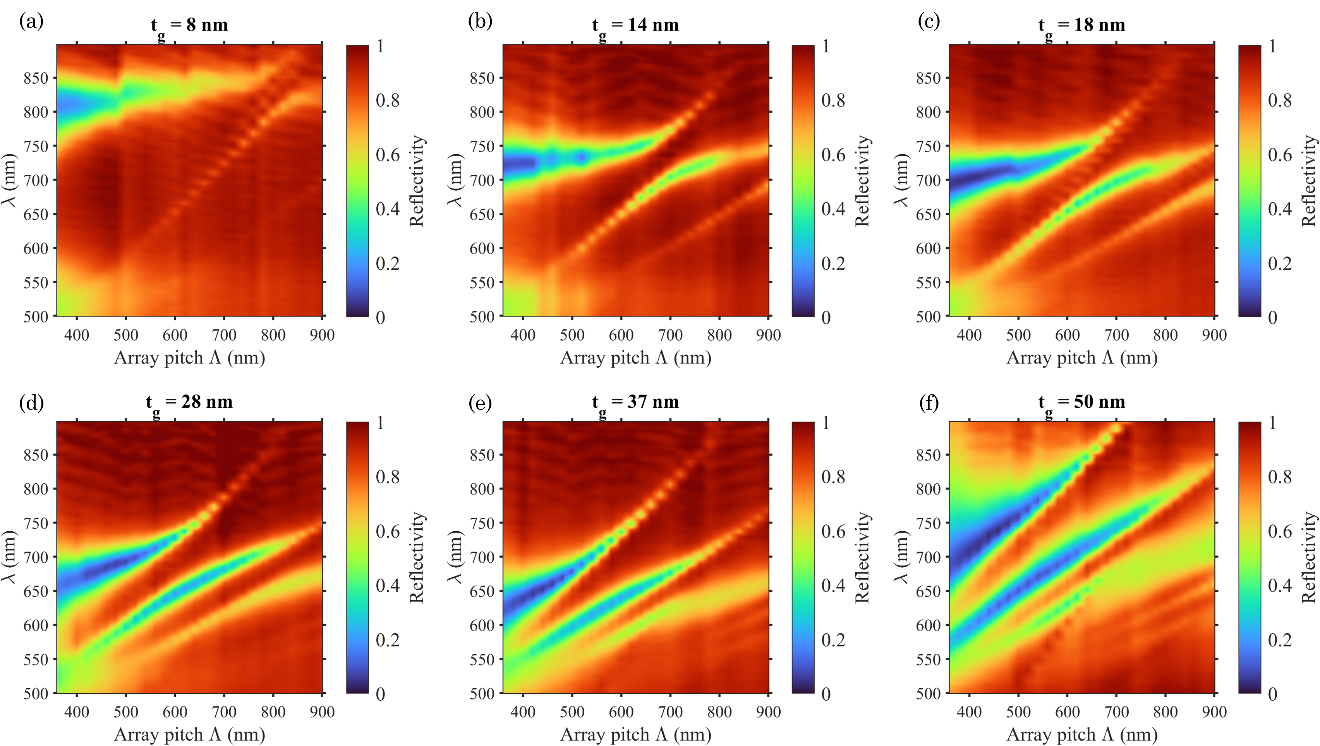


Figure D4: Experimental measurements of reflection spectra of gold NPoM arrays as a function of array pitch for different gap thicknesses, at a fixed nanodisk diameter of d = 83 nm.


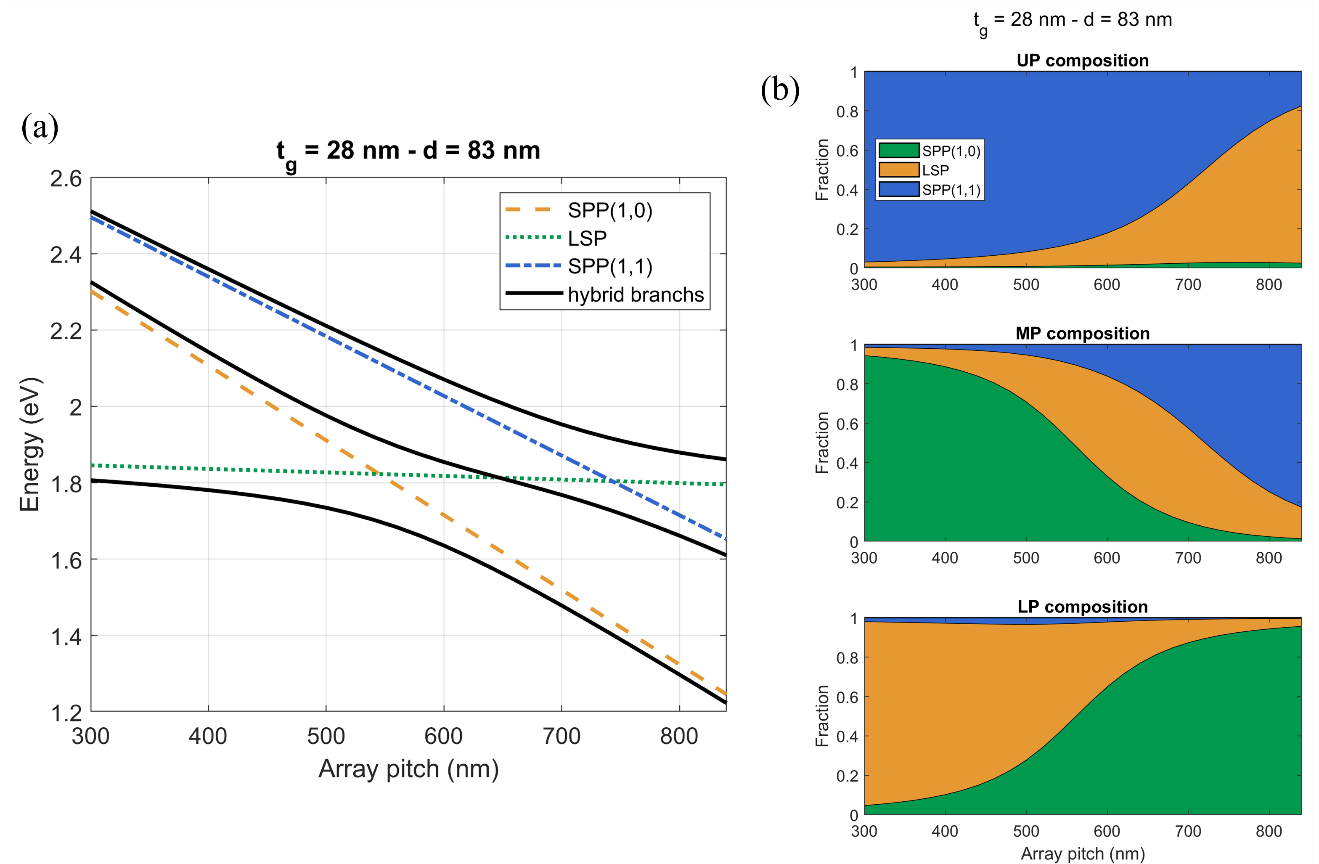


Figure D5: Hopfield coefficient analysis of the hybrid modes for the geometry (t_g_ = 28 nm – d = 83 nm) (a) dispersion of the fitted hybrid and uncoupled modes (b) Hopfield fractions for each branch of the hybrid modes.

# Dephasing time measurements

The ITR-PEEM results are fitted by applying a model with a single damped harmonic oscillator for pitches $\Lambda=\left\{ 460,520 \right\} nm$ and two coupled damped harmonic oscillators for $\Lambda=\left\{ 620,660,740 \right\} nm$, as detailed in [1]. Complementary to Figure 5 in the main text, pitches $\Lambda=\left\{ 520,620 \right\} nm$ are presented in Figure E3.

The local electric field in Eq. (1) from the main text is expressed as a convolution of the incident electric field of the form $E_{I}\left( t,\tau\right)=E_{0}\left( t \right)+E_{0}\left( t+\tau\right)$ and a response function $R\left( t \right)$ of the NPoM array given by

$$\begin{aligned} E_{loc}\left( t,\tau\right)=E_{I}\left( t,\tau\right)*R\left( t \right)=\int_{-\infty}^{\infty} d\omega E_{0}\left( \omega\right)R(\omega)(1+e^{-i\omega\tau})e^{i\omega t}\#\left( S1 \right) \end{aligned}$$

where we use the experimentally measured spectrum $I\left( \lambda\right)$ and thus $E_{0}\left( \omega\right)=\sqrt{I(\omega)}$. In Figure E1, we show the experimental FROG-trace and experimentally determined pulse duration.

The response function $R\left( \omega\right)$ of the NPoM array for a single damped harmonic oscillator is given by:

$$\begin{aligned} R\left( \omega\right)=\left[ \omega_{r}^{2}+2\gamma i\omega-\omega^{2} \right]^{-1}\#\left( S2 \right) \end{aligned}$$

with resonance frequency $\omega_{r}$ and damping factor $\gamma$.

For two coupled harmonic oscillators, the response function $R\left( \omega\right)$ of the NPoM array is given by a linear combination of each coupled mode $R_{1}\left( \omega\right)$ and $R_{2}\left( \omega\right)$:

$$\begin{aligned} R\left( \omega\right)=a_{1}R_{1}\left( \omega\right)+a_{2}R_{2}(\omega)\#\left( S3 \right) \end{aligned}$$

where

$$\begin{aligned} R_{1}\left( \omega\right)=\frac{-\omega_{LP}-2\gamma_{LP}i\omega+\omega^{2}}{g^{2}-(\omega_{LP}^{2}-2\gamma_{LP}i\omega+\omega^{2})(\omega_{MP}^{2}-2\gamma_{MP}i\omega+\omega^{2})}\#\left( S4 \right) \end{aligned}$$

and

$$\begin{aligned} R_{2}\left( \omega\right)=\frac{g}{g^{2}-(\omega_{LP}^{2}-2\gamma_{LP}i\omega+\omega^{2})(\omega_{MP}^{2}-2\gamma_{MP}i\omega+\omega^{2})}\#\left( S5 \right) \end{aligned}$$

for resonances $\omega_{LP,MP}$, damping factors $\gamma_{LP,MP}$ and coupling constant $g$.

Figure E4 presents the experimental ITR-PEEM results for all pitches with the corresponding fit overlaid. The fit results by finding the collective minimum of squared difference between experimental data and model given by

$$\begin{aligned} F\left( \tau\right)=\sum_{k} \left| S_{PE}\left( \tau_{k} \right)-S\left( \tau_{k} \right) \right|^{2} \#\left( S6 \right) \end{aligned}$$

using global optimization methods. The dephasing times are extracted according to $T_{j}=\frac{2}{\gamma_{j}}$, where $j\in\{LP,MP\}$.


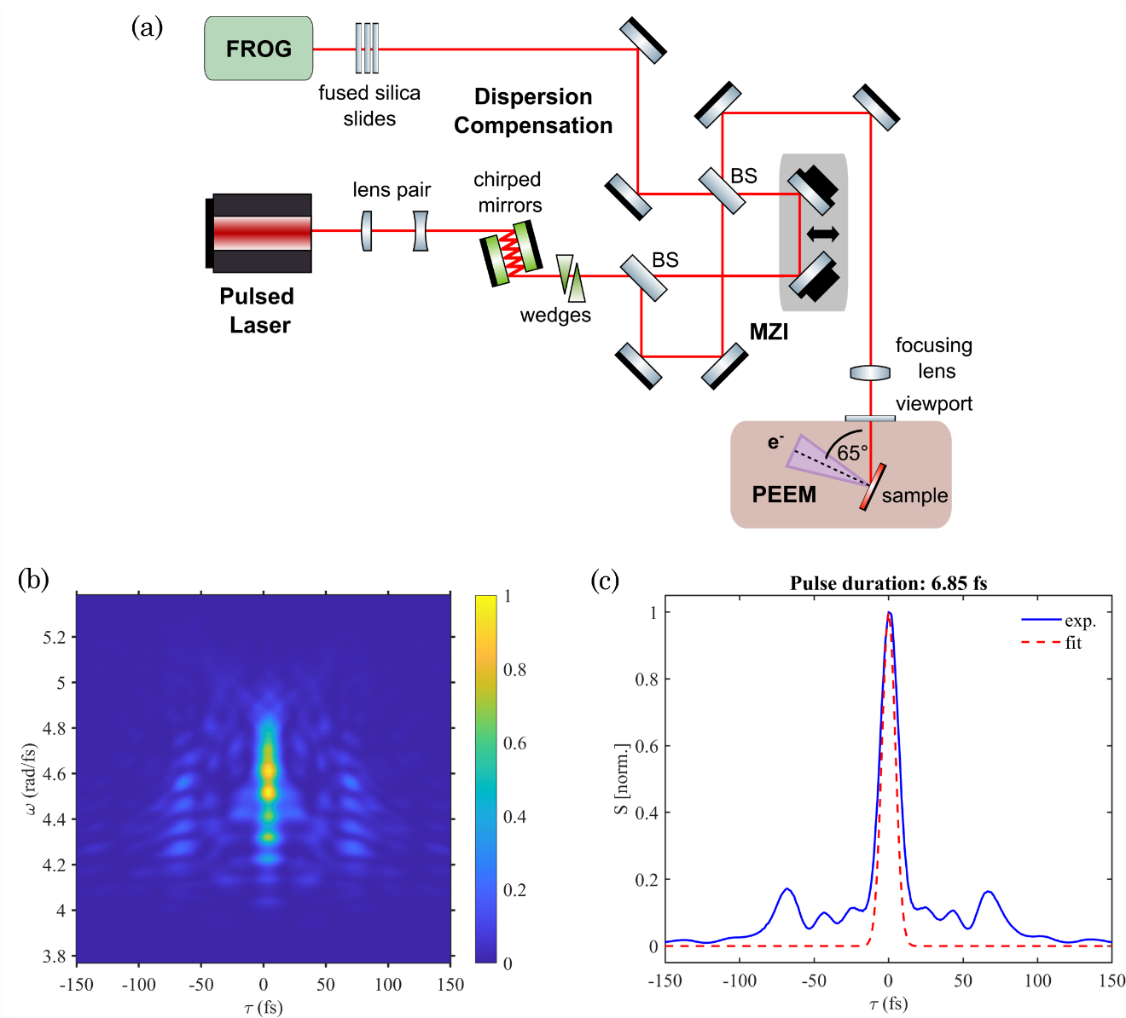


Figure E1: Pulse characterization for ITR-PEEM with in-house built FROG setup. (a) Schematic of the ITR-PEEM setup (b) FROG-trace of dispersion compensated pulse (c) Pulse duration of about 6.85 fs results by fitting the intensity autocorrelation (blue curve) with a Gaussian (red curve).


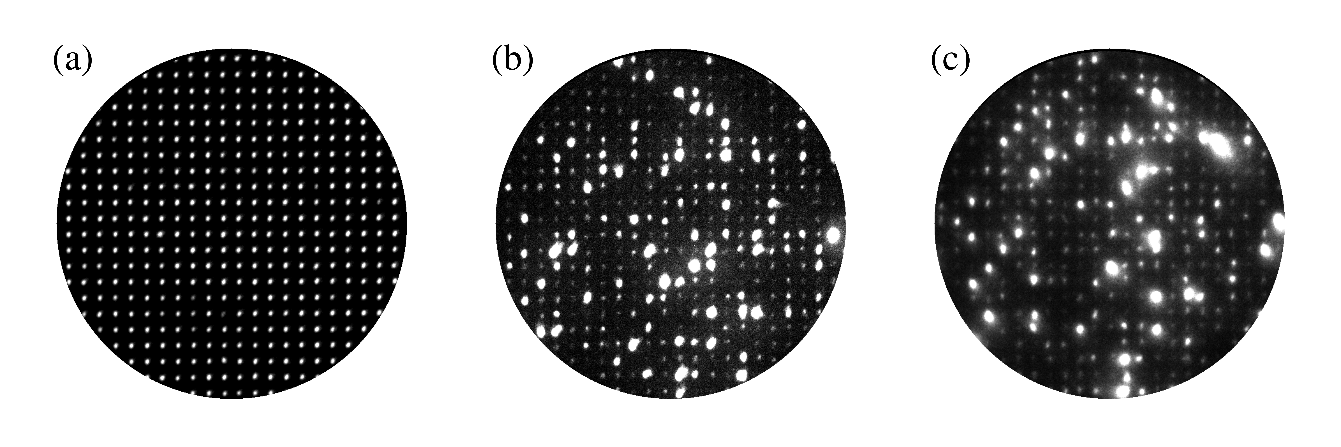


Figure E2: Exemplary PEEM images for configuration $\Lambda=460 nm$ with different light sources using 11µm field-of-view: (a) Hg discharge lamp. (b) Output of sub-6 femtosecond laser oscillator constructively interfered ($\tau=0 fs$). (c) Output of separate femtosecond laser oscillator with center wavelength set to 805 nm. (b-c) High aspect ratio features, like sharp edges, locally enhance the electric field, creating bright spots. While this affects the photoemission (PE) yield in specific areas, overall absorption remains unchanged.


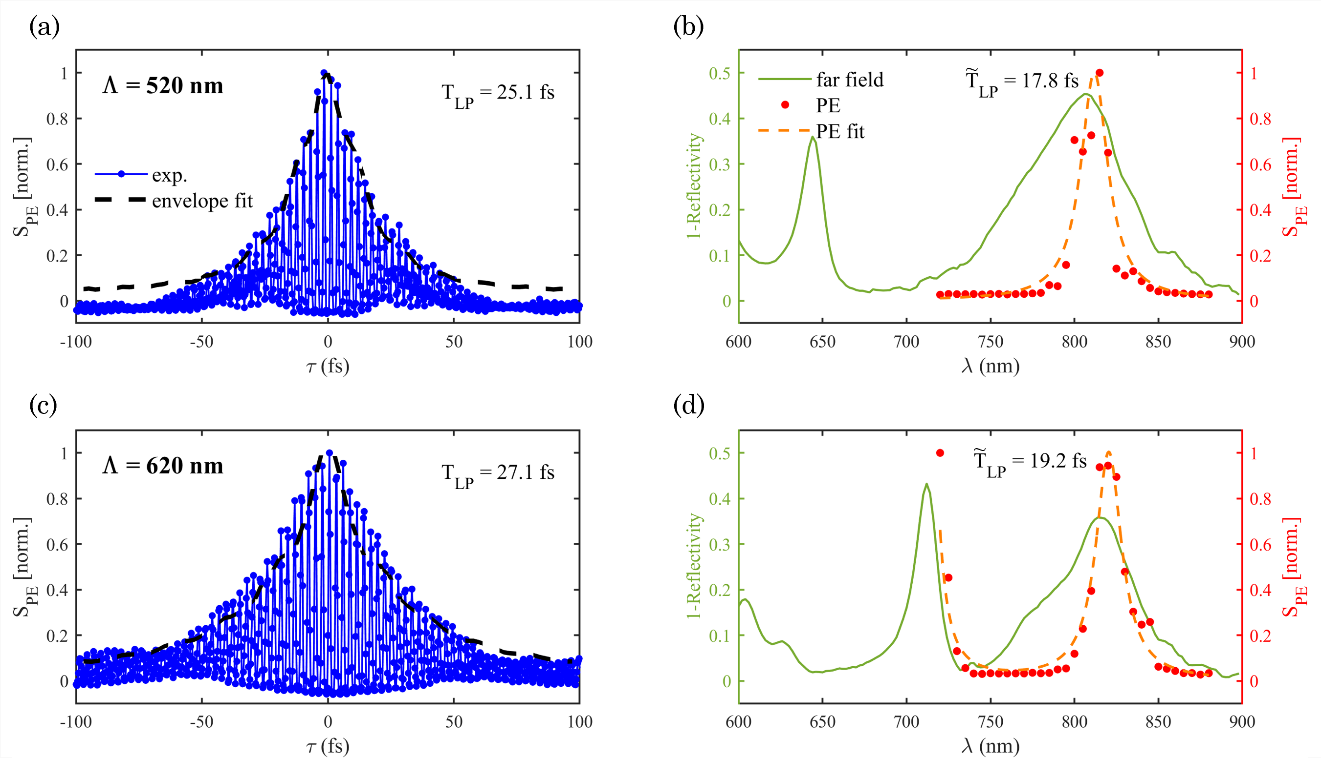


Figure E3: Additional ITR-PEEM and excitation-wavelength depending PE yield results for pitches $\Lambda=\left\{ 520,620 \right\} nm$.


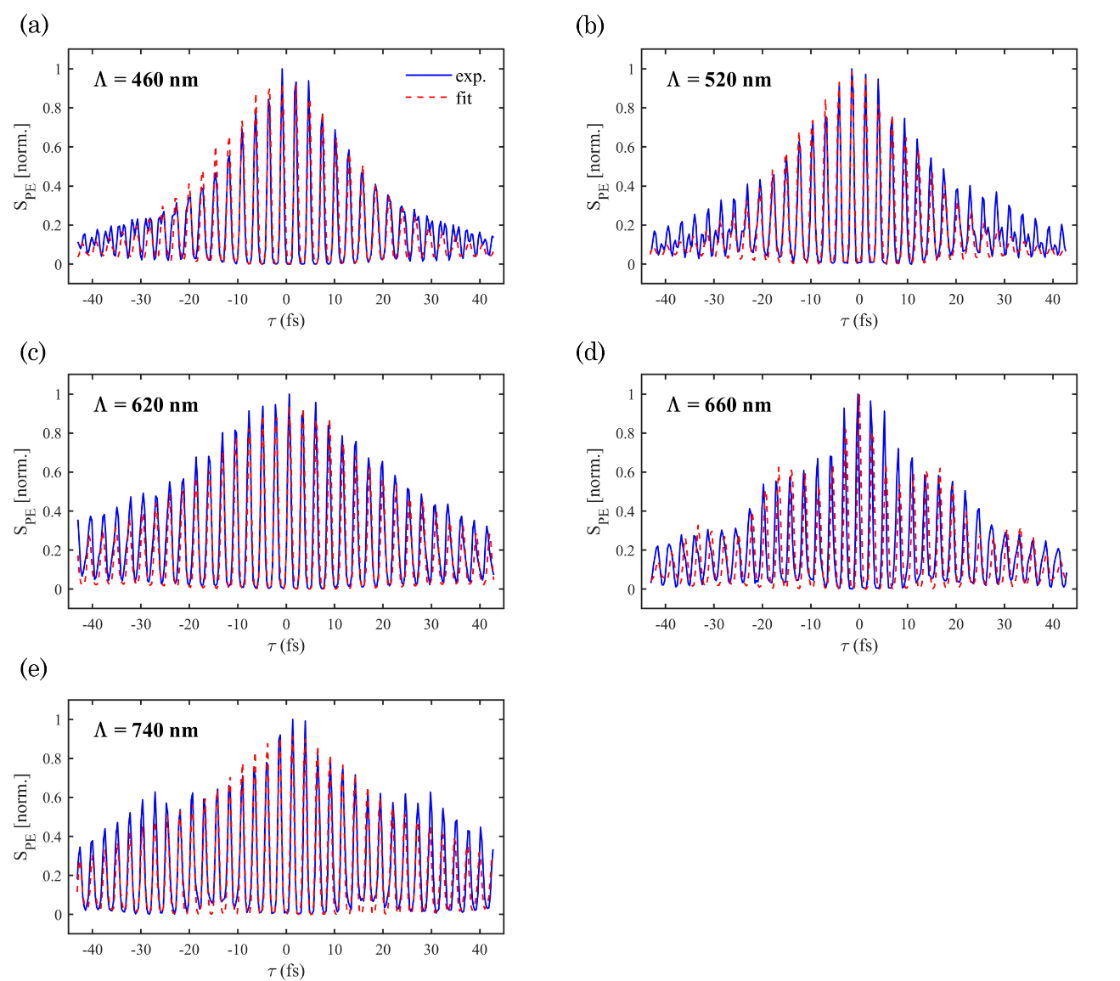


Figure E4: Fit of experimental ITR-PEEM data. (a,b) are fitted with the single and (c,d,e) with the coupled oscillator model.

# Reference

[1] M. Aeschlimann *et al.*, “Determination of local optical response functions of nanostructures with increasing complexity by using single and coupled Lorentzian oscillator models,” *Appl. Phys. B*, vol. 122, no. 7, p. 199, Jul. 2016, doi: 10.1007/s00340-016-6471-3.
